# A robust culture system to generate neural progenitors with gliogenic competence from clinically relevant induced pluripotent stem cells for treatment of spinal cord injury

**DOI:** 10.1002/sctm.20-0269

**Published:** 2020-11-23

**Authors:** Yasuhiro Kamata, Miho Isoda, Tsukasa Sanosaka, Reo Shibata, Shuhei Ito, Toshiki Okubo, Munehisa Shinozaki, Mitsuhiro Inoue, Ikuko Koya, Shinsuke Shibata, Tomoko Shindo, Morio Matsumoto, Masaya Nakamura, Hideyuki Okano, Narihito Nagoshi, Jun Kohyama

**Affiliations:** ^1^ Department of Physiology Keio University School of Medicine Tokyo Japan; ^2^ Department of Orthopedic Surgery Keio University School of Medicine Tokyo Japan; ^3^ Regenerative & Cellular Medicine Kobe Center Sumitomo Dainippon Pharma Co., Ltd Kobe Japan

**Keywords:** HLA‐homo, induced pluripotent stem cells, neural stem progenitor cells, oligodendrocyte, remyelination, spinal cord injury

## Abstract

Cell‐based therapy targeting spinal cord injury (SCI) is an attractive approach to promote functional recovery by replacing damaged tissue. We and other groups have reported the effectiveness of transplanting neural stem/progenitor cells (NS/PCs) derived from human induced pluripotent stem cells (hiPSCs) in SCI animal models for neuronal replacement. Glial replacement is an additional approach for tissue repair; however, the lack of robust procedures to drive iPSCs into NS/PCs which can produce glial cells has hindered the development of glial cell transplantation for the restoration of neuronal functions after SCI. Here, we established a method to generate NS/PCs with gliogenic competence (gNS/PCs) optimized for clinical relevance and utilized them as a source of therapeutic NS/PCs for SCI. We could successfully generate gNS/PCs from clinically relevant hiPSCs, which efficiently produced astrocytes and oligodendrocytes in vitro. We also performed comparison between gNS/PCs and neurogenic NS/PCs based on single cell RNA‐seq analysis and found that gNS/PCs were distinguished by expression of several transcription factors including *HEY2* and *NFIB*. After gNS/PC transplantation, the graft did not exhibit tumor‐like tissue formation, indicating the safety of them as a source of cell therapy. Importantly, the gNS/PCs triggered functional recovery in an SCI animal model, with remyelination of demyelinated axons and improved motor function. Given the inherent safety of gNS/PCs and favorable outcomes observed after their transplantation, cell‐based medicine using the gNS/PCs‐induction procedure described here together with clinically relevant iPSCs is realistic and would be beneficial for SCI patients.

## Graphical abstract

Our study demonstrated a successful and robust procedure to generate neural stem progenitor cells (NS/PCs) with gliogenic competence from clinically relevant induced pluripotent stem cells, which in turn efficiently differentiated into astrocytes and oligodendrocytes in vitro and in vivo. These findings indicate that our method to produce gliogenic NS/PCs will be useful for developing cell‐based therapies for spinal cord injury.

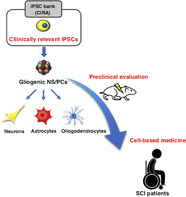

**Significance statement**

This study demonstrated a successful and robust procedure to generate neural stem/progenitor cells (NS/PCs) with gliogenic competence from clinically relevant human induced pluripotent stem cells, which in turn efficiently differentiated into astrocytes and oligodendrocytes in vitro and in vivo. Transplantation of the NS/PCs triggered remyelination and functional recovery in a spinal cord injury (SCI) animal model. These findings indicate that this method to produce gliogenic NS/PCs will be useful for developing cell‐based therapies for SCI.

## 1 INTRODUCTION

The central nervous system (CNS) has a limited capacity to regenerate; therefore, any loss of function by traumatic injury is mostly irreversible. Spinal cord injury (SCI) is a traumatic injury to the CNS that results in severe physical impairments, including permanent paralysis, sensory disturbance, and bladder dysfunction.^1^ The number of new cases annually is approximately 120 000 in the United States and 5000 in Japan.^2^, ^3^ However, approaches to treat SCI patients are currently limited.

In recent years, cell transplantation therapies are considered to be effective in SCI animal models, and preclinical evaluations have been initiated.^4^, ^5^, ^6^, ^7^, ^8^, ^9^, ^10^ As a cell source for the therapies, human induced pluripotent stem cells (hiPSCs) have attracted particular interest due to a limited number of ethical concerns, as they are directly generated from somatic cells.^11^ We have reported the efficacy of cell transplantation in SCI animal models using neural stem/progenitor cells (NS/PCs) derived from hiPSCs and the integration of hiPSC‐derived neurons into the host neuronal networks.^12^, ^13^, ^14^, ^15^ However, mouse gliogenic NS/PCs were reported to produce more favorable outcomes than neurogenic NS/PCs after transplantation into a SCI animal model, indicating additional processes mediated by gliogenic NS/PCs can be therapeutic in addition to neuronal replacement by grafted NS/PCs.^16^ Moreover, transplantation of NS/PCs derived from myelin‐deficient *shiverer* mice, produced less functional recovery after transplantation into injured spinal cord, indicating the importance of remyelination in the repair process by grafted NS/PC‐derived oligodendrocytes.^17^ These results indicate the importance of glial cells including oligodendrocytes for treatment of SCI. Since hiPSCs can serve as an unlimited and scalable source of somatic cells, successful derivation of glial cells would benefit a broad range of neurological disorders including SCI. However, it is noteworthy that hiPSC‐NS/PCs predominantly differentiate into neurons both in vitro and in vivo.^12^, ^13^, ^18^ Accordingly, numerous attempts have been made to improve the generation of glial cells including oligodendrocytes from hiPSCs.^19^, ^20^, ^21^, ^22^, ^23^ However, these procedures often required long duration to generate glial cells and/or complicated techniques.^19^, ^20^, ^24^ Moreover, most procedures are optimized for iPSCs generated in individual laboratories. There is limited information for reproducibility of differentiation over multiple iPSCs. Therefore, a standardized protocol to generate glial cells from iPSCs has not been developed.

In addition, there is another issue to be in account. Numerous studies have demonstrated the potency of iPSCs in regenerative medicine; however, most of these studies used iPSCs that were not generated for clinical use. Generation and validation of clinically relevant iPSCs is costly and time‐consuming, which hinders the preclinical evaluation of iPSCs. Thus, there is a need to develop a cell bank of iPSCs, which provides qualified iPSCs to researchers. Then, each researcher would conduct preclinical evaluation of iPSC‐derivatives without generating and validating iPSCs by himself. The Center for iPS Cell Research and Application (CiRA) at Kyoto University, a core center for iPSC research in Japan, was funded by the Japanese government to prepare clinical grade iPSCs from donors who were homozygous for the three major human leucocyte antigen (HLA) loci haplotypes (HLA‐A, HLA‐B, and HLA‐DR) (HLA‐homozygous), which would reduce immune rejection after allogenic transplantation.^25^, ^26^ However, there is no established protocols to generate NS/PCs optimized for therapeutic intervention, and, most importantly, their safety and therapeutic efficacy after transplantation remain unknown.

**FIGURE 1:**
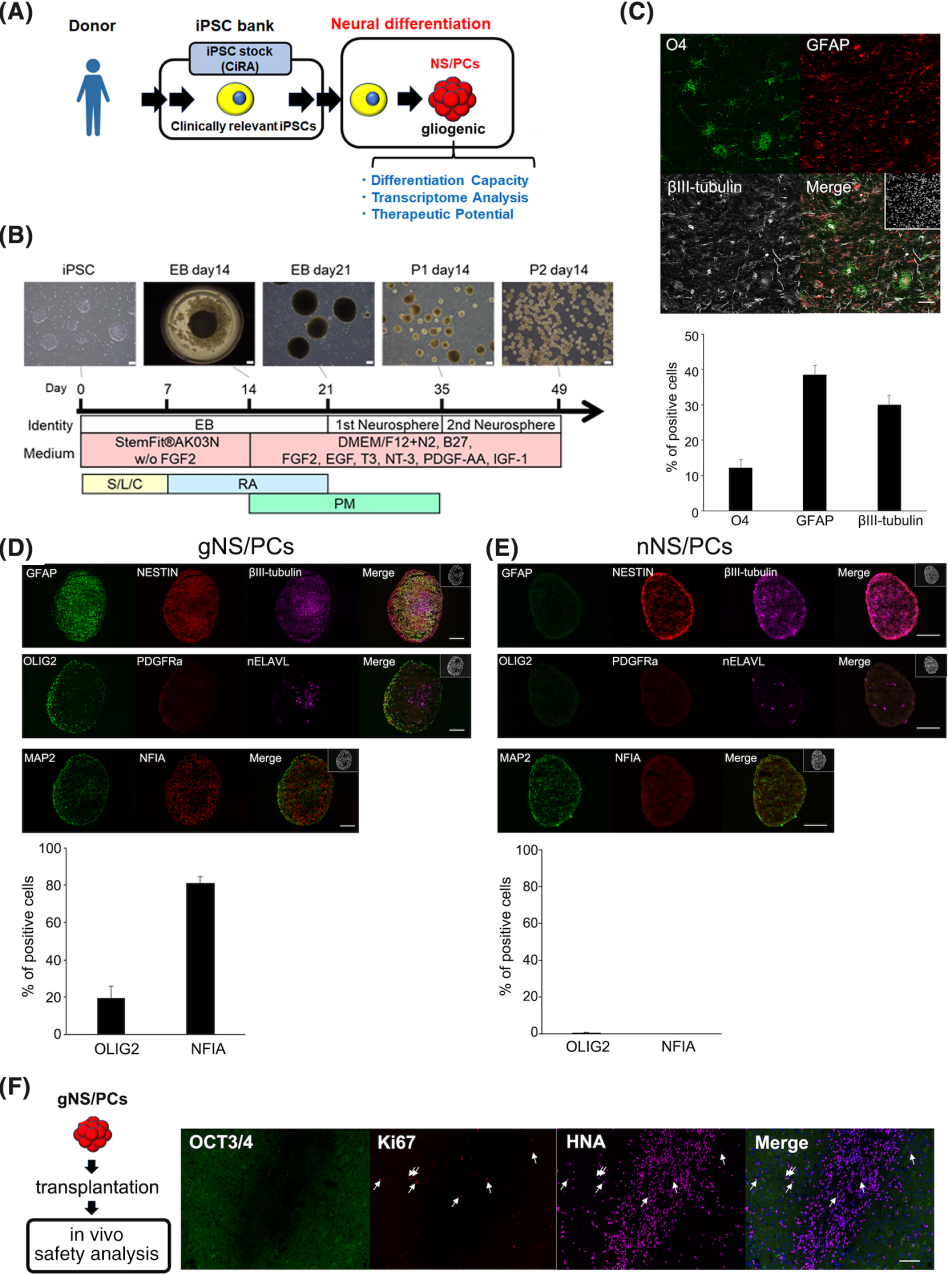
Differentiation of iPSCs into glia‐enriched NS/PCs. A, Study overview. A clinically relevant iPSC stock from a human leucocyte antigen (HLA)‐homozygous donor sample had been generated at the Center for iPS Cell Research and Application (CiRA). The provided iPSCs were processed for neural differentiation to generate NS/PCs with gliogenic competence. Differentiation capacity, transcriptome analysis, and therapeutic potential of the NS/PCs were analyzed. iPSC, induced pluripotent stem cell. B, Schematic illustration of the protocol for generating NS/PCs with gliogenic competence (gNS/PCs) from hiPSCs with representative images of each differentiation step. EB, embryoid body; P, passage; S, SB431542; L, LDN193189; C, CHIR99021; RA, retinoic acid; PM, purmorphamine. Scale bar = 200 μm. C, Representative immunocytochemistry images after 31 days of differentiation of third passage (P3) neurospheres using neuronal (βIII‐tubulin), astrocyte (GFAP), and oligodendrocyte (O4) markers. Scale bar = 50 μm. Quantification is shown in the lower panel (means ± SEM for n = 3 independent biological replicates). D, Representative immunocytochemistry images of gNS/PC neurospheres (P3, 77DIV) using cell type‐specific markers for neural progenitors (NESTIN), neurons (MAP2, nELAVL, and βIII‐tubulin), glia‐committed neural progenitors (NFIA), astrocytes (GFAP), and oligodendrocyte progenitors (PDGFRα and OLIG2). Nuclei are counterstained with Hoechst 33342. Scale bars = 100 μm. Quantification of the frequency of marker‐positive cells is shown in the lower panel (means ± SEM for n = 3 independent biological replicates). E, Representative immunocytochemistry images of nNS/PC neurospheres (P6, 63DIV) using cell type‐specific markers for neural progenitors (NESTIN), neurons (MAP2, nELAVL, and βIII‐tubulin), glia‐committed neural progenitors (NFIA), astrocytes (GFAP), and oligodendrocyte progenitors (PDGFRα and OLIG2). Nuclei are counterstained with Hoechst 33342. Scale bars = 100 μm. Quantification of the frequency of marker‐positive cells is shown in the lower panel (means ± SEM for n = 3 independent biological replicates). F, Assessment of tumorigenicity of gNS/PCs in vivo. Tumorigenicity and ongoing proliferation were assessed using OCT3/4 and Ki67 at 185 days after transplantation into immunodeficient animals; None of cells were positive for OCT3/4 and 2.22% ± 0.26% were positive for Ki67 among the grafts (n = 2 independent biological replicates). gNS/PCs, NS/PCs with gliogenic competence. Scale bar = 100 μm. GFAP, glial fibrillary acidic protein; hiPSCs, human induced pluripotent stem cells; NS/PCs, neural stem/progenitor cells

Here, we aimed to establish and optimize a robust protocol to produce NS/PCs with gliogenic competence (gNS/PCs) from clinically relevant iPSCs, from which oligodendrocytes can be generated. We also assessed the efficacy and safety of the NS/PCs in a rodent model of SCI (Figure 1A). This study is the first evaluation of clinically relevant HLA‐homozygous iPSCs in an SCI model.

## 2 MATERIALS AND METHODS

See further details in the Supporting Information Materials and Methods.

### 2.1 Cell culture and neural induction

The hiPSC lines (WJ14s01, WJ14s02, WJ23s01, and WJ23s02) were established at CiRA from umbilical cord blood cells obtained from HLA‐homozygous healthy donors (homozygous for HLA‐A*24:02; HLA‐B*52:01; HLA‐DRB1*15:02 haplotypes) under xeno‐free and feeder‐free conditions via the transduction of reprogramming factors (OCT3/4, SOX2, KLF4, L‐MYC, dominant‐negative p53, and EBNA1) using episomal vectors.^27^, ^28^ Most experiments were performed using WJ14s01. Other lines were used for validation of the protocol to generate gNS/PCs as indicated in the manuscript. hiPSCs were maintained by the feeder‐free iPSC culture method.^27^ For gNS/PCs induction, hiPSCs were dissociated into single cells using TrypLE Select (Thermo Fisher Scientific) and reaggregated to form embryoid bodies (EBs) using 96‐well low cell‐adhesion plates (Sumitomo Bakelite) at a density of 9000 cells/well in StemFit AK03N (Ajinomoto) without fibroblast growth factor 2 (FGF2) supplemented with 100 nM LDN193189, 3 μM SB431542, and 3 μM CHIR99021 (all from Stemgent) at 37°C and 5% CO_2_/5% O_2_ for 7 days. On day 7, the medium was switched to StemFit AK03N without FGF2 supplemented with 1 μM retinoic acid (RA, Sigma). The medium was changed every day. On day 14, EBs were transferred into ultralow attachment culture flasks (Corning) and cultured in proliferation medium supplemented with 1 μM RA and 1 μM purmorphamine (PM, Merck Millipore) at 37°C and 5% CO_2_/20% O_2_. Proliferation medium is a mixture of DMEM/F12 (Wako) containing 1% N2 (Thermo Fisher Scientific), 2% B‐27 without Vitamin A (Thermo Fisher Scientific), 60 ng/mL triiodo‐L‐thyronine (T3, Sigma), 10 ng/mL Platelet‐derived growth factor (PDGF)‐AA(PeproTech), 20 ng/mL FGF2 (PeproTech), 10 ng/mL epidermal growth factor (EGF, PeproTech), 10 ng/mL insulin growth factor 1 (IGF‐1, R&D systems), and 10 ng/mL neurotrophin 3 (NT‐3, R&D Systems). On day 21, EBs were enzymatically dissociated into single cells using TrypLE Select, and the dissociated cells were cultured in suspension at a density of 1.0 × 10^5^ cells/mL in proliferation medium supplemented with 1 μM PM to generate the first neurospheres. The medium was changed every 7 days. After 14 days, the first neurospheres were dissociated in the same manner as described above and cultured in proliferation medium without PM. Passaging was carried out once every 14 to 28 days thereafter. The third passage neurospheres were used for transplantation. Neuronal and glial differentiation was performed as previously described.^23^ Undissociated 15 to 20 neurospheres were plated on 8‐well chamber glass slides coated with 1% growth factor reduced Matrigel (Corning), and cultured in differentiation medium that consisted of KBM Neural Stem Cell (KOHJIN BIO) supplemented with 2% B27, 1% nonessential amino acids (Thermo Fisher Scientific), 60 ng/mL T3, 10 ng/mL NT‐3, 10 ng/mL Leukemia Inhibitory Factor (Millipore), and 25 ng/mL Ciliary Neurotrophic Factor (Peprotech) for 4 weeks in a humidified atmosphere of 5% CO_2_. Half of the medium was changed every 2 or 3 days. The neurogenic NS/PCs were kindly provided by Dr Yonehiro Kanemura and cultured as previously described.^29^

### 2.2 SCI animal model and feeder‐free hiPSCs (ffiPSC)‐gNS/PCs transplantation

Eight‐week‐old female nonobese diabetic‐severe combined immunodeficiency mice (Charles River Laboratories) were anesthetized with an intraperitoneal injection of ketamine (100 mg/kg) and xylazine (10 mg/kg). Contusive SCI was induced at the level of the tenth thoracic spinal vertebra using an Infinite Horizon impactor (60‐70 kdyn; Precision Systems and Instrumentation), as described previously.^30^ After SCI, 12.5 mg/kg ampicillin was administered intramuscularly. Seven days after injury, all the mice were evaluated for motor function, and mice with a Basso Mouse Scale (BMS)^31^ score of 2.5 or higher were excluded from the study. The SCI mice were randomly assigned to each group (15 mice per group). For gNS/PCs group mice, 9 days after injury, ffiPSC‐gNS/PCs (5 × 10^5^ cells in 2 μL of phosphate‐buffered saline [PBS]) were transplanted into the lesion epicenter of each mouse with a Hamilton syringe with a 28 G metal needle using a microstereotaxic injection system (KDS310; Muromachi‐Kikai Co., Ltd.). An equal volume of PBS was similarly injected into PBS group mice in place of the ffiPSC‐gNS/PCs. The injected depth was 0.3 to 0.8 mm and injection speed was 1 μL/min. A mouse of gNS/PCs group and three mice of PBS group died during the observation period and were excluded from the study. For evaluation of tumorigenicity of ffiPSC‐gNS/PCs, intracerebral ffiPSC‐gNS/PCs transplantation was performed with stereotactic injection of 1.0 × 10^6^ cells in 4 μL of PBS through a 22 G needle into the striatum of 22‐week‐old female F344/NJcl‐rnu/rnu rats (Clea Japan) (1 mm anterior and 3 mm lateral from bregma and 5.5 mm below the brain surface) and injection speed was 6 μL/min.

### 2.3 Motor function analyses

Hind limb locomotor function was evaluated for 12 weeks after transplantation using the BMS. At 12 weeks after transplantation, a rotating rod apparatus, a treadmill gait analysis, and a kinematics were performed. Detailed methods are described in the Supporting Information.

### 2.4 Quantification and statistical analyses

Statistical analyses were performed using SPSS version 25 (Japan IBM). All data are reported as the mean ± SEM. The normality of the distribution of data points was verified using Shapiro‐Wilk test. The Mann‐Whitney *U* test was used for H&E staining (control group, n = 6; ffiPSC‐gNS/PCs group, n = 6), Luxol fast blue (LFB) staining (control group, n = 6; ffiPSC‐gNS/PCs group, n = 6), myelin basic protein (MBP) area (control group, n = 6; ffiPSC‐gNS/PCs group, n = 6), biotin dextran amine (BDA)‐labeled RtST (control group, n = 6; ffiPSC‐gNS/PCs group, n = 6), body weight gain and loss (control group, n = 12; ffiPSC‐gNS/PCs group, n = 14), kinematic analyses (control group, n = 5; ffiPSC‐gNS/PCs group, n = 8), Treadmill gait analysis (control group, n = 12; ffiPSC‐gNS/PCs group, n = 14), rotarod test (control group, n = 12; ffiPSC‐gNS/PCs group, n = 14), and H‐reflex (control group, n = 9; ffiPSC‐gNS/PCs group, n = 7). Repeated‐measures two‐way analysis of variance (ANOVA), followed by the Turkey‐Kramer test, was used for the BMS analysis (control group, n = 12; ffiPSC‐gNS/PCs group, n = 14). In each case, *P < .05 and **P < .01 were statistically significant.

## 3 RESULTS

### 3.1 Generation of glial progenitor‐enriched NS/PCs from feeder‐free HLA‐homozygous hiPSCs

To realize clinical application of NS/PCs derived from iPSCs, we focused on clinically relevant feeder‐free hiPSCs (ffiPSCs) provided by iPSC bank in CiRA.^25^, ^26^ These iPSCs had been established from HLA‐homozygous donors to lower the immunogenicity of iPSC‐derivatives^28^, ^32^ (Figure 1A). Since neural differentiation protocols usually depend on the innate differentiation capacity of the individual iPSC clones and are successful for a limited number of iPSC clones,^33^ we were motivated to modify previously reported neural induction procedures for NS/PCs, which had been examined over multiple iPSC clones established from different somatic tissue origins.^29^, ^34^ Given that the NS/PCs generated from this procedure were less gliogenic,^29^ we attempted to determine experimental conditions to generate NS/PCs containing glial progenitors using the ffiPSCs. Initially, we applied this protocol on the ffiPSCs derived from umbilical cord blood cells (WJ14s01) and examined differentiation capacity of NS/PCs. As shown in Figure S1, we successfully differentiated ffiPSCs into NS/PCs which mainly produced neurons and, as expected, glial differentiation capacity was limited: any oligodendrocytes judged by the expression of oligodendrocyte marker O4 was not detected. Then, we modified this protocol to the trigger precocious glial differentiation from NS/PCs and determined the protocols as follows. Among various cytokines and/or chemical compounds, we chose CHIR99021 (CHIR), a GSK‐3β inhibitor,^35^ and RA for astrocyte‐generation to accelerate differentiation of NS/PCs, as previously reported.^23^, ^36^, ^37^, ^38^, ^39^ As shown in Figure 1B, we exposed ffiPSCs to CHIR and dual Smad inhibitors for 7 days, followed by treatment with RA for 14 days. To enhance oligodendrocyte differentiation, we chose purmorphamine (PM), a Sonic hedgehog agonist that ventralizes neural tissues, to provide a permissive environment for oligodendrocytes,^40^, ^41^, ^42^ and added growth factors and cytokines (triiodo‐L‐thyronine [T3], neurotrophin 3 [NT‐3], Platelet‐derived growth factor [PDGF]‐AA, IGF‐1, FGF2, and EGF) which were reported to be useful for oligodendrocyte generation from day 14 to day 35.^23^ Under this procedure, the NS/PCs were generated in neurosphere‐based floating conditions (Figure 1B).

We applied this protocol to the ffiPSCs and obtained neurospheres, which would contain NS/PCs. To examine the differentiation capacity of the NS/PCs, third passage (P3) neurospheres were plated for terminal differentiation and assessed by immunocytochemistry using cell type‐specific markers for neurons (βIII‐tubulin), astrocytes (glial fibrillary acidic protein [GFAP]), and oligodendrocytes (O4). As shown in Figure 1C, neurospheres at P3 exhibited efficient differentiation potency: 12.21% ± 2.30% had differentiated into O4^+^ oligodendrocytes, 38.63% ± 2.59% into GFAP^+^ astrocytes, and 30.03% ± 2.64% into βIII‐tubulin^+^ neurons. To further characterize the NS/PCs, the P3 neurospheres were directly processed for immunocytochemistry. As shown in Figure 1D, the neurospheres highly expressed a neural progenitor marker, NESTIN. The neurospheres also expressed an astrocytic marker (GFAP) and neuronal markers (βIII‐tubulin, nELAVL, and MAP2). Notably, the neurospheres also expressed a gliogenic progenitor marker, NFIA, and oligodendrocyte‐committed markers, including OLIG2 and platelet‐derived growth factor receptor α (PDGFRα). Among them, NFIA and OLIG2 were of interests given their essential role for generation of astrocytes and oligodendrocyte, respectively.^41^, ^43^, ^44^, ^45^ OLIG2 and NFIA were highly expressed in the neurospheres (19.67% ± 6.32% and 81.26% ± 3.36%, respectively), indicating that the current procedures enabled to convert iPSCs to NS/PCs with gliogenic capacity. In contrast, consistent with the neuron‐biased differentiation capacity of the iPSC‐derived NS/PCs,^29^ these neurogenic NS/PCs exhibited expression of NESTIN and βIII‐tubulin and nELAVL while expression of OLIG2 and NFIA in the NS/PCs were almost neglectable (0.54% ± 0.23% and 0%, respectively) (Figure 1E). Taken together, these observations indicated that the NS/PCs prepared using the current protocol contained high numbers of NS/PCs which could produce glial cells; thus, we designated the NS/PCs derived from ffiPSCs as NS/PCs with gliogenic competence (ffiPSC‐gNS/PCs).

We further applied the protocol to three additional clones of ffiPSCs (WJ14s02, WJ23s01, and WJ23s02) and consistently obtained NS/PCs that produced astrocytes and oligodendrocytes, indicating that the procedure was robust enough to generate NS/PCs from the ffiPSCs (data not shown).

Tumorigenicity is one of potential barriers to put iPS‐derived cells into clinical setting.^46^ Accordingly, we examined the safety of ffiPSC‐gNS/PCs by transplanting them into the striatum of immunodeficient rats as previously described^34^, ^47^ (Figure 1F). We analyzed the graft about 6 months after the transplantation and detected no visible tumorigenic mass in the transplanted immunodeficient animals (data not shown). Since some iPSC‐NS/PCs have been reported to exhibit tumorigenicity with the expression of OCT3/4 (a marker for pluripotent stem cells),^48^ we further evaluated the histological features of the grafts by immunostaining with antibodies against OCT3/4 and Ki67 (a marker for proliferative cells). Although some cells were still positive for Ki67, we detected no OCT3/4‐expressing cells in the graft (Figure 1F). Therefore, we found that ffiPSC‐gNS/PCs were less tumorigenic.

### 3.2 Transcriptome signature of ffiPSC‐gNS/PCs

**FIGURE 2:**
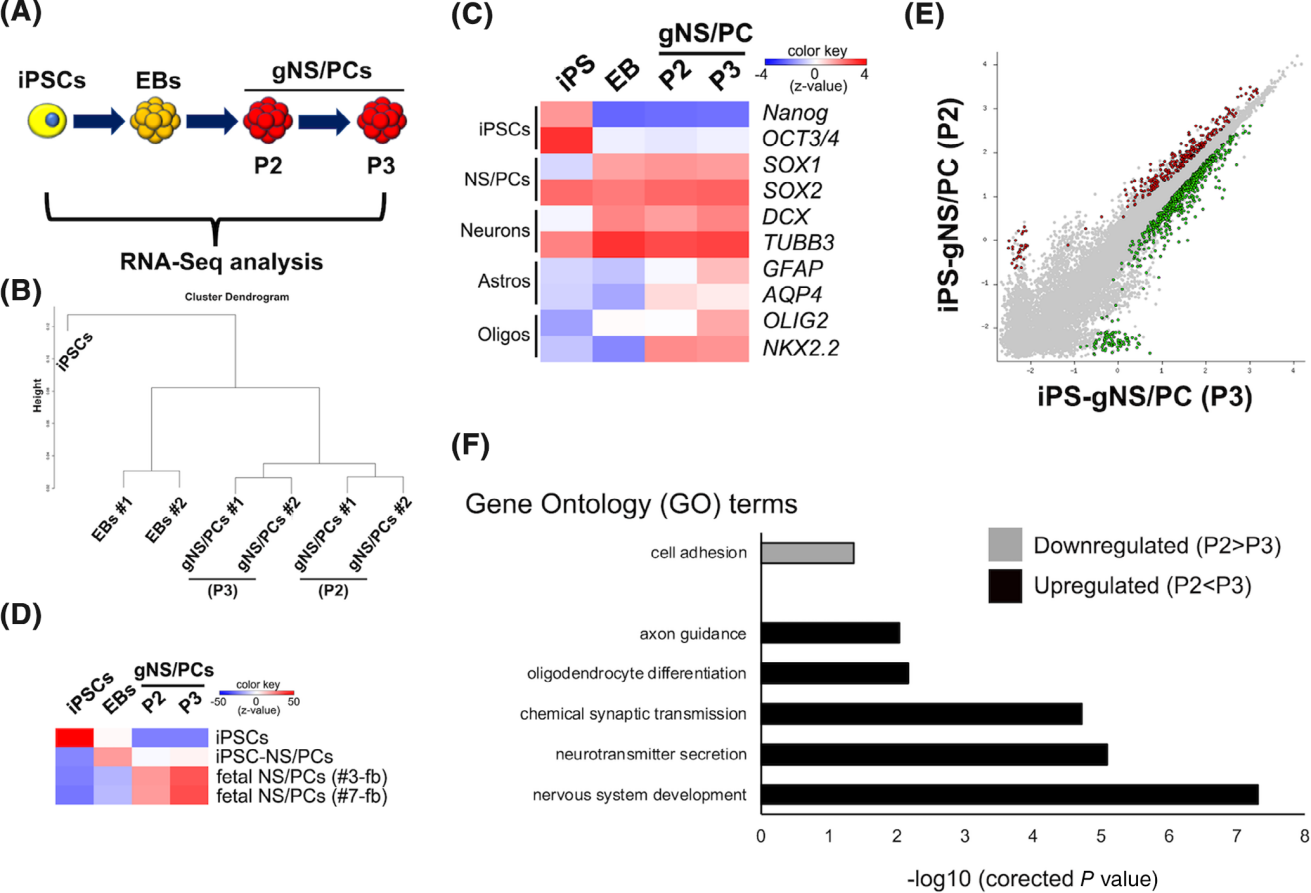
Transcriptomic characterization of feeder‐free hiPSCs (ffiPSC)‐gNS/PCs. A, Transcriptome analysis of ffiPSCs (iPSCs), ffiPSC‐EBs (EBs), and ffiPSC‐gNS/PCs (gNS/PCs) (P2 and P3) by RNA‐seq. B, Dendrogram clustering of iPSCs, EBs, and gNS/PCs (P2 and P3) based on global gene expression. Samples were taken from two independent experiments to generate gNS/PCs from ffiPSCs (#1 and #2). C, Heatmap displaying normalized expression (z‐score) of iPSC‐specific (*NANOG* and *OCT3/4*), NS/PC‐specific (*SOX1* and *SOX2*), neuron‐specific (*DCX* and *TUBB3*), astrocyte‐specific (*GFAP* and *AQP4*), and oligodendrocyte‐specific (*OLIG2* and *NKX2.2*) genes in iPSCs, EBs, and gNS/PCs (P2 and P3). D, Correlation analysis of gene expression in iPSCs, EBs, and gNS/PCs with the gene expression profiles of the indicated cell types (iPSCs, iPSC‐NS/PCs, human fetal NS/PCs). The published gene expression profiles of iPSCs, iPSC‐NS/PCs, and human fetal NS/PCs were loaded from GSE76900. E, A scatter plot was used to assess differences in gene expression between gNS/PCs (P2) and gNS/PCs (P3). The genes labeled green (306 genes) or red (714 genes) have a >1.5‐fold change in expression between the two groups. F, GO analysis of downregulated and upregulated genes in P3 gNS/PCs compared to P2 gNS/PCs. Enrichment of corresponding GO terms is shown. EBs, embryoid bodies; gNS/PCs, neural stem/progenitor cells with gliogenic competence; GO, gene ontology; iPSCs, induced pluripotent stem cells

To characterize the ffiPSC‐gNS/PCs, we performed RNA‐seq analysis on ffiPSCs, ffiPSC‐derived EBs, and ffiPSC‐gNS/PCs (P2 and P3) (Figure 2A). Hierarchical clustering analysis of the transcriptional profiles of each sample exhibited clear separation between cell types (Figure 2B). To clarify the transcriptional difference, we examined the expression of cell type‐specific marker genes for iPSCs (*NANOG* and *OCT3/4*), NS/PCs (*SOX1* and *SOX2*), neurons (*DCX* and *TUBB3*), astrocytes (*GFAP* and *AQP4*) and oligodendrocytes (*OLIG2* and *NKX2.2*) in the current samples. As shown in Figure 2C, iPSC markers were downregulated, while genes specific for NS/PCs and neurons were upregulated and maintained in EBs and ffiPSC‐gNS/PCs. In contrast, genes associated with astrocytes and oligodendrocytes were gradually upregulated in the gNS/PCs, indicating that ffiPSC‐gNS/PCs acquire the capacity to differentiate into glial cells during culture from EBs to gNS/PCs. Importantly, these genes were further upregulated from P2 to P3 ffiPSC‐gNS/PCs. We further cross‐referenced our data set with the expression profiles of other types of neural progenitors, including neurogenic iPSC‐NS/PCs^12^, ^13^, ^18^ and tripotent human fetal NS/PCs which produce astrocytes and oligodendrocytes in addition to neurons^49^ using Exatlas (https://lgsun.grc.nia.nih.gov/exatlas/). As expected, the gene expression profile of ffiPSC‐gNS/PCs was similar to that of human fetal NS/PCs while that of EBs was more similar to neurogenic iPSC‐NS/PCs (Figure 2D). Notably, the trend of the gNS/PCs transcriptome becoming more similar to that of human fetal NS/PCs was apparent during cellular passage, revealing a transcriptional transition in ffiPSC‐gNS/PCs from P2 to P3. We selected differentially expressed genes between these cells (306 upregulated and 714 downregulated genes in P3 ffiPSC‐gNS/PCs) (Figure 2E) and performed Gene Ontology (GO) analysis to illuminate the biological functions (Figure 2F). While the GO term “cell adhesion” was identified in the downregulated gene set, GO terms obtained from upregulated genes included “nervous system development,” “neurotransmitter secretion,” “chemical synaptic transmission,” and “axon guidance,” indicating neuronal maturation during passaging. In addition, “oligodendrocyte differentiation” was a notable term identified as it may explain the maturation of NS/PCs toward glial lineages.

**FIGURE 3:**
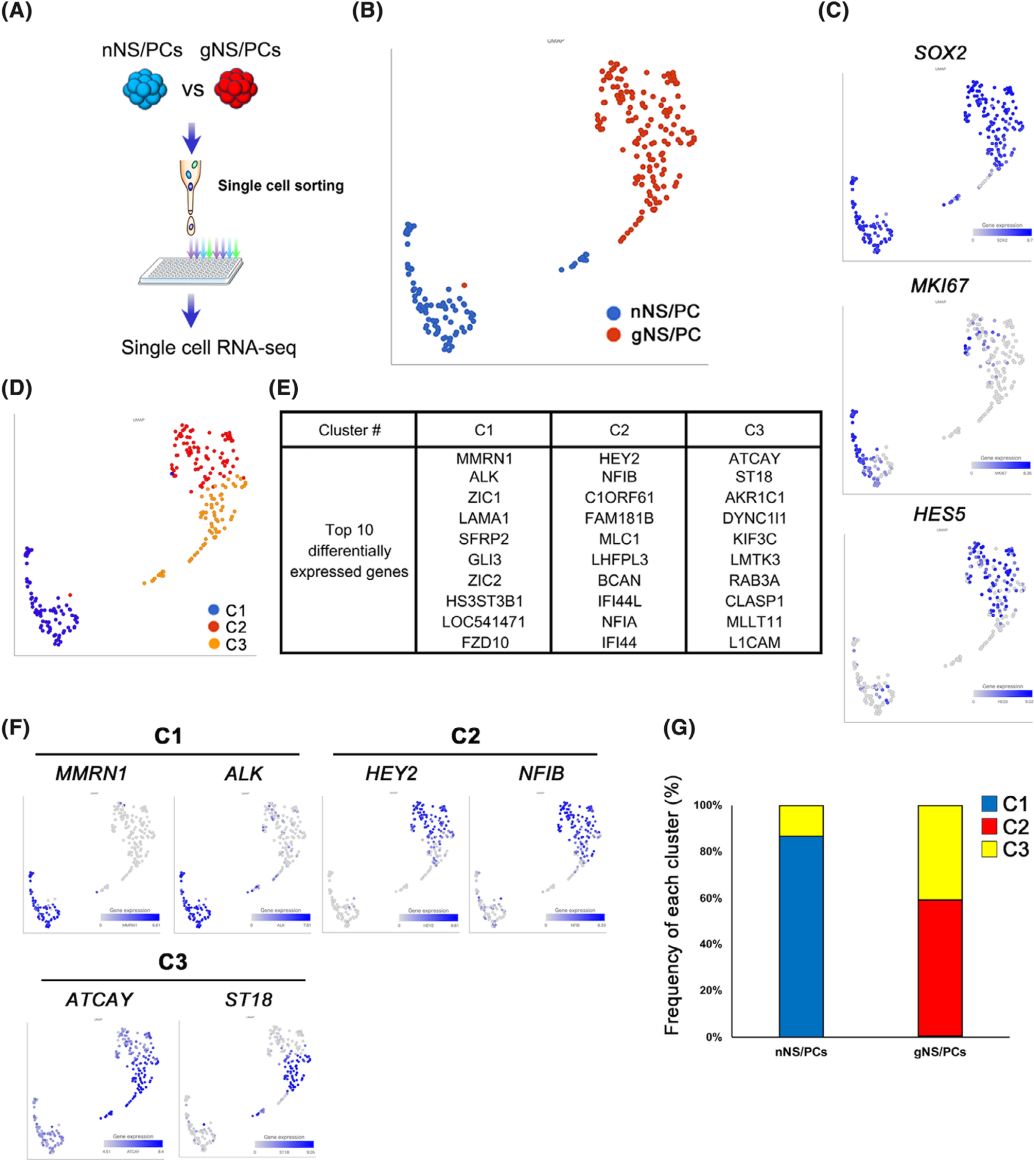
Single cell RNA‐seq analysis of feeder‐free hiPSCs (ffiPSC)‐gNS/PCs. A, Experimental paradigm for single cell RNA‐seq analysis for neurogenic ffiPSC‐NS/PCs (ffiPSC‐nNS/PCs) and ffiPSC‐gNS/PCs. After single cell‐sorting into 96 well plate, a procedure for single cell RNA‐Seq analysis was performed. B, Uniform manifold approximation and projection (UMAP) plot generated from scRNA‐seq data of 241 cells, 91 of which were ffiPSC‐nNS/PCs and 150 were ffiPSC‐gNS/PCs. Blue dots represent ffiPSC‐nNS/PCs, while red dots represent ffiPSC‐gNS/PCs. C, UMAPs displaying the expression of the indicated genes. D, UMAP plot displaying cell clusters generated by the graph‐based clustering method. E, The top 10 differentially expressed genes in each cluster (C1‐C3) were shown. F, UMAPs displaying the expression of representative differentially expressed genes in each cluster. G, Proportion of clusters (C1‐C3) in ffiPSC‐nNS/PCs and ffiPSC‐gNS/PCs. gNS/PCs, neural stem/progenitor cells with gliogenic competence

To further characterize ffiPSC‐gNS/PCs, we performed single‐cell RNA‐seq (scRNA‐seq) analysis of P3 ffiPSC‐gNS/PCs. As a reference, we used neurogenic iPSC‐NS/PCs derived from the same parental iPSCs (ffiPSC‐nNS/PCs)^29^ (Figures S1 and 3A). We sorted the NS/PCs into 96‐well plates and processed them for scRNA‐seq analysis.^50^ After quality control, 150 ffiPSC‐gNS/PCs and 91 ffiPSC‐nNS/PCs were further analyzed. Cell‐clustering analysis by uniform manifold approximation and projection revealed clear separation of ffiPSC‐nNS/PCs and ffiPSC‐gNS/PCs (Figure 3B). The differential distribution of the clusters between these NS/PCs suggested that each group was substantially different at the transcriptome level (Figure 3B). For example, both NS/PCs shared similar *SOX2* expression profiles; however, *MKI67*, a marker for proliferation, was mainly expressed in ffiPSC‐nNS/PCs, indicative of the progressive expandable capacity of neurogenic NS/PCs (Figure 3C). Since activation of Notch signaling is essential to acquire gliogenic potential of NS/PCs during mouse CNS development,^51^ we examined expression of *HES5*, a downstream effector of Notch signaling, and found that *HES5* was mainly observed in the ffiPSC‐gNS/PCs, suggesting gliogenic properties of ffiPSC‐gNS/PCs (Figure 3C). Given that in our procedure to generate ffiPSC‐gNS/PCs required RA, well‐known for caudalizing activity on neural tissues during development,^39^ we examined difference in the regional identity. As shown in Figure S2, we examined expression of regional specific neural markers^52^, ^53^, ^54^ and observed ffiPSC‐gNS/PCs hold regional properties as caudalized neural tissues compared to ffiPSC‐nNS/PCs, indicating regional identity in addition to differentiation capacity was altered. To characterize difference between these NS/PCs, we classified them based on transcriptome, and identified three clusters (C1‐C3) (Figure 3D). Each cluster was characterized by marker genes that were visualized by the expression of selected cluster‐defining gene sets (Figure 3E,F). Within the clusters, characteristic expression of neuronal genes, including *ATCAY* ^55^ and *ST18*,^56^ was observed in cluster C3 (Figure 3E,F). Furthermore, Cluster C1 was characterized by expression of an early neuroectodermal marker, *MMRN1*.^57^ Frequency of cells harboring the features of Cluster C1 was high in ffiPSC‐nNS/PCs, consistent with the high neurogenic capacity of ffiPSC‐nNS/PCs^29^ (Figure 3G). In contrast, the expression of glial genes, including *HEY2* and *NFIB*, was observed in Cluster C2 (Figure 3E,F). Interestingly, the Cluster C2 was highly enriched in ffiPSC‐gNS/PCs, further supporting the glia‐committed properties of ffiPSC‐gNS/PCs compared with ffiPSC‐nNS/PCs (Figure 3G).

### 3.3 Transplanted ffiPSC‐gNS/PCs survive, migrate, and differentiate into three neural lineages in vivo

**FIGURE 4:**
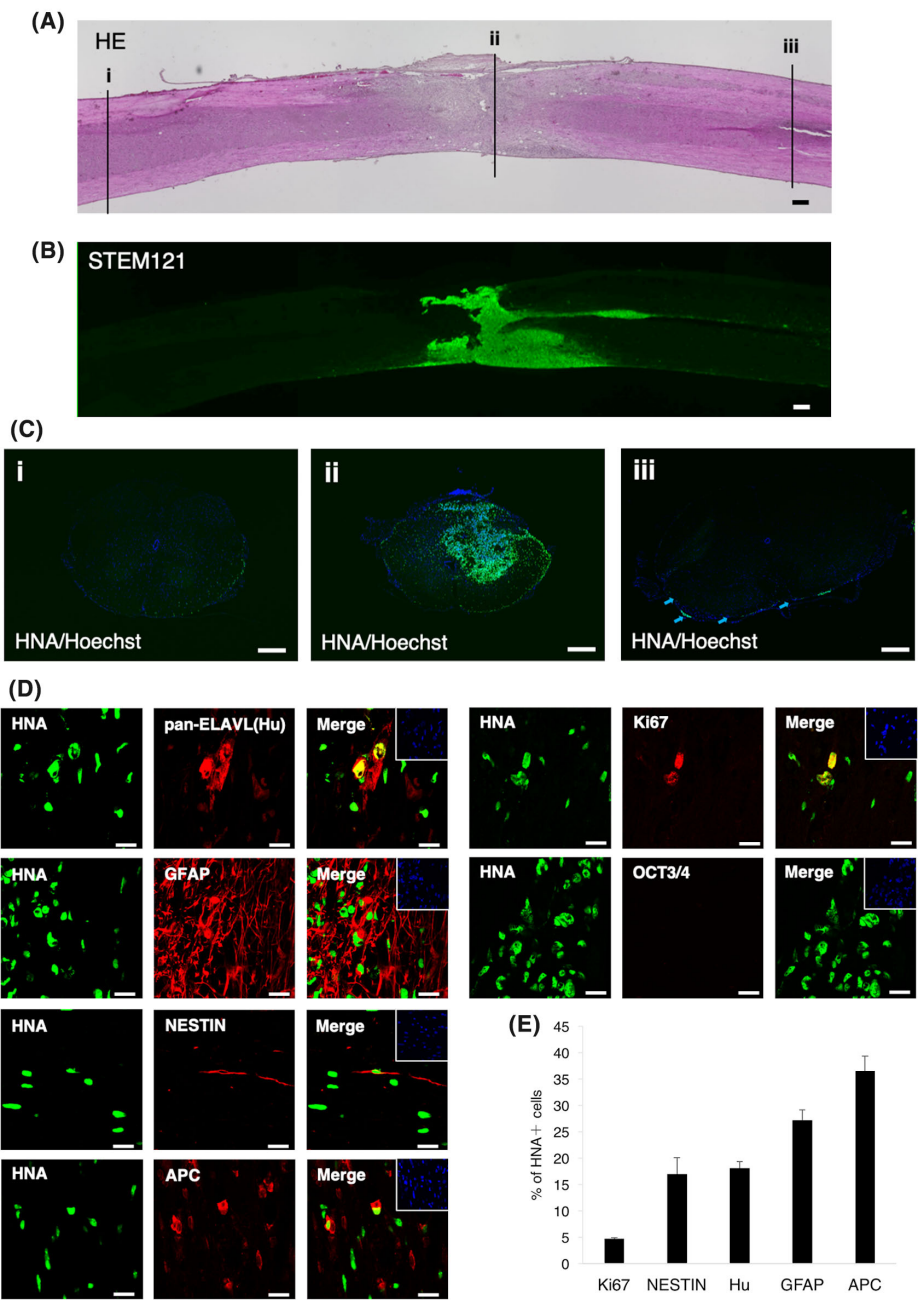
Differentiation potential of feeder‐free hiPSCs (ffiPSC)‐gNS/PCs after transplantation. A, Representative H&E‐stained images of sagittal sections of the spinal cord 12 weeks after transplantation. Positions of three axial section images in (C) are indicated with numbered lines. Scale bar = 200 μm. B, The distribution of transplanted cells was visualized by STEM121 immunoreactivity. The survival of transplanted cells at the lesion epicenter was confirmed. Scale bar = 200 μm. C, Human nuclear antigen (HNA) staining of axial sections at positions indicated in (A). The HNA^+^ grafted cells were integrated at the lesion epicenter (ii) and migrated rostrally (i) and caudally (iii). Scale bars = 200 μm. D, Characterization of the transplanted cells was determined using the cell type‐specific markers indicated in the figure. Representative images of HNA^+^ grafted cells together with Ki67, OCT3/4, NESTIN, pan‐ELAVL (Hu), GFAP, and adenomatous polyposis coli (APC) staining. Insets: Hoechst nuclear staining of each field. Scale bars = 20 μm. E, Quantification of the frequency of cell type‐specific marker‐positive cells among HNA^+^ transplanted cells 12 weeks after transplantation. GFAP, glial fibrillary acidic protein; gNS/PCs, neural stem/progenitor cells with gliogenic competence

We next examined whether the NS/PCs could promote functional recovery after CNS injury. We generated an SCI animal model by inducing contusive SCI with moderate severity at the 10th thoracic vertebrae level in adult female immunodeficient mice.^14^, ^30^, ^58^ 5.0 × 10^5^ ffiPSC‐gNS/PCs were transplanted into the center of the lesion 9 days after the SCI. First, to investigate any configuration changes of the injured spinal cord, we examined the cross‐sectional area of the injured spinal cord by H&E staining 12 weeks after transplantation (Figure S3A). The transverse area of the spinal cord at the lesion epicenter was significantly larger in the ffiPSC‐gNS/PCs group than in the PBS group (Figure S3B). Furthermore, there was no visible tumor‐like tissue formation observed in any of the H&E‐stained images from mice in the ffiPSC‐gNS/PCs group (data not shown). To further clarify the distribution and location of the transplanted cells, we performed immunostaining using antibodies specific to human cytoplasm (STEM121) and human nuclear antigen (HNA) 12 weeks after transplantation. The transplanted cells labeled with STEM121 survived and were widely spread throughout the injured spinal cord (Figure 4A,B). To examine the location of the graft‐derived cells, we visualized the cells with HNA‐specific antibodies and examined the distribution of the cells in axial sections of the spinal cord. We found HNA^+^ cells from the epicenter to +4 mm rostrocaudal in the ffiPSC‐gNS/PCs group, indicating that the transplanted cells themselves migrated (Figure 4C). To further evaluate the capacity of differentiation of the NS/PCs, we examined the coexpression of HNA and cell type‐specific markers at 12 weeks after transplantation, beginning with OCT3/4 and Ki67 for evaluation of tumorigenicity of the NS/PCs.^48^ Similar to the results observed in ffiPSC‐gNS/PCs‐transplanted striatum (Figure 1F), although 4.71% ± 0.20% of the HNA^+^ cells were positive for Ki67, there were no OCT3/4‐expressing cells in the grafts (Figure 4D,E), indicating additional evidence of the safety of the ffiPSC‐gNS/PCs. As predicted by the presence of Ki67^+^ proliferative cells, 17.02% ± 3.10% HNA^+^ cells were positive for NESTIN, indicating remnant proliferating progenitors was contained in the grafts (Figure 4D,E). Furthermore, by using cell type specific makers for neurons (pan‐ELAVL [Hu]), astrocytes (GFAP), and oligodendrocytes (APC), the transplanted ffiPSC‐gNS/PCs differentiated into the three neural lineages: 18.12% ± 1.22% had differentiated into pan‐ELAVL (Hu)^+^ neurons, 27.23% ± 1.92% into GFAP^+^ astrocytes, and 36.56% ± 2.82% into APC^+^ oligodendrocytes (Figure 4E).

### 3.4 ffiPSC‐gNS/PCs‐derived mature oligodendrocytes contribute to remyelination

**FIGURE 5:**
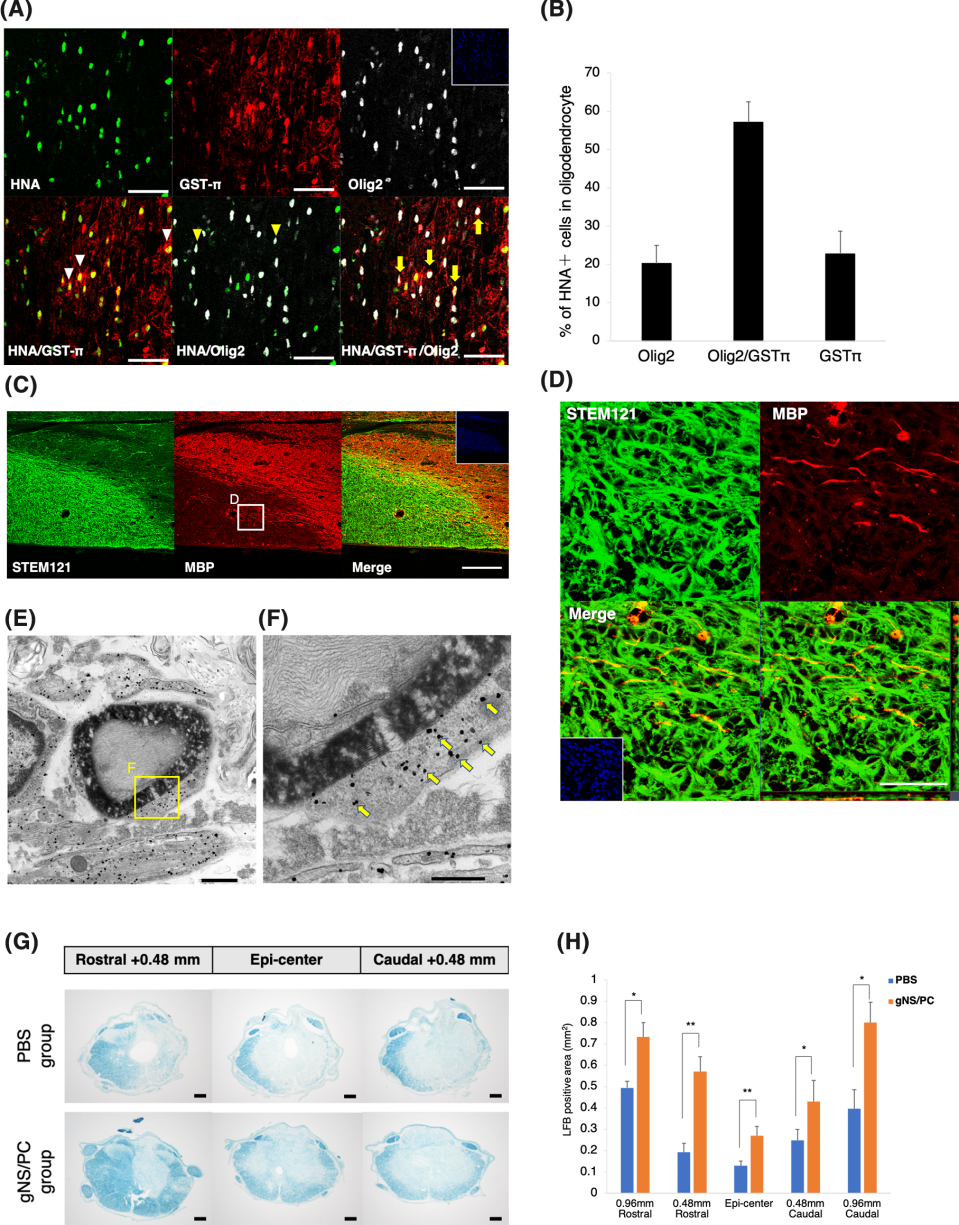
Contribution of transplanted feeder‐free hiPSCs (ffiPSC)‐gNS/PCs‐derived oligodendrocytes to the remyelination of spared axons in the injured spinal cord. A, Representative images of HNA^+^ grafted cells together with OLIG2 and GST‐π staining. Nuclei are counterstained with Hoechst 33342. White arrowheads indicate HNA^+^/GST‐π^+^ cells. Yellow arrowheads indicate HNA^+^/OLIG2^+^ cells. Yellow arrows indicate HNA^+^/OLIG2^+^/GST‐π^+^ cells. Scale bars = 50 μm. B, Quantification of the frequency of oligodendrocyte‐lineage cells among HNA^+^ transplanted cells at 12 weeks after transplantation. C,D, Representative images of sagittal sections stained for STEM121 and MBP. Higher magnification of boxed region in (C) is shown in (D). White arrowheads indicate the STEM121^+^/MBP^+^ area. Scale bars = (C): 200 μm, (D): 50 μm. E,F, Representative images of immunoelectron microscopy. Grafted cells were detectable by the black dots observed upon STEM121 antibody staining (arrows). The STEM121^+^ dots were often observed in the outer cytoplasm of the myelin sheath. Scale bars = (E): 1000 nm, (F): 500 nm. G, Representative LFB‐stained images of axial sections at the lesion epicenter and at sites 0.48 mm rostral and caudal in the PBS and ffiPSC‐gNS/PCs groups. LFB, luxol fast blue. Scale bars = 200 μm. H, Quantitative analysis revealed that the LFB‐stained area in the axial sections was significantly larger in the ffiPSC‐gNS/PCs group than in the PBS group at the lesion epicenter and each site rostral and caudal. Values are means ± SEM (control group, n = 6; ffiPSC‐gNS/PCs group, n = 6; **P < .01). gNS/PCs, neural stem/progenitor cells with gliogenic competence; PBS, phosphate‐buffered saline

The capacity of ffiPSC‐gNS/PCs to differentiate into an oligodendrocyte lineage was of interest because it is usually less abundant to observe oligodendrocyte differentiation in grafted iPS‐NS/PCs.^12^, ^14^ Furthermore, remyelination of injured neurons is a desirable therapeutic intervention in SCI.^17^, ^59^ Thus, the contribution of ffiPSC‐gNS/PCs on remyelination after SCI were further assessed. First, to characterize the extent of oligodendrocyte differentiation from ffiPSC‐gNS/PCs, we performed immunohistochemistry using antibodies against OLIG2 and GST‐π (markers for immature and mature oligodendrocytes, respectively). Within the oligodendrocyte‐lineage cells, we found OLIG2^+^/GST‐π^−^ (immature oligodendrocytes), OLIG2^+^/GST‐π^+^ (committed oligodendrocytes), and OLIG2^−^/GST‐π^+^ cells (mature oligodendrocytes) in the grafts (Figure 5A,B). Importantly, 22.73% ± 5.98% of the ffiPSC‐gNS/PCs‐derived oligodendrocyte‐lineage cells were matured OLIG2^−^/GST‐π^+^ oligodendrocytes at 12 weeks after transplantation. Next, we further examined oligodendrocyte differentiation of ffiPSC‐gNS/PCs by immunohistochemistry using MBP and found that there were several STEM121^+^/MBP^+^ areas in the ffiPSC‐gNS/PCs group (Figure 5C,D). Immunoelectron microscopy showed that myelin sheaths were strongly associated with myelin cytoplasm with nanogold‐labeled STEM121^+^ spots (Figure 5E,F). These results indicate that transplanted ffiPSC‐gNS/PCs‐derived oligodendrocytes formed mature myelin sheaths on spared axons. To examine the myelinated region in cross‐sections of the spinal cord, we visualized myelin sheaths in the injured spinal cord by LFB staining at 12 weeks after transplantation. The LFB^+^ area was dramatically reduced in the injured spinal cord, indicative of demyelination in the SCI model (Figure 5G). In contrast, quantitative myelinated areas compared with the PBS group from the epicenter to +0.96 mm rostrocaudal (Figure 5H). Consistently, we found that MBP^+^ areas in the transplanted group were also significantly larger than those of the PBS group (Figure S4A,B). Taken together, these results indicate that these NS/PCs harbor the capacity to differentiate into mature oligodendrocytes and form myelin sheaths.

Since reconstitution of neuronal circuit is important for functional recovery after SCI^14^ and we observed neuronal differentiation of ffiPSC‐gNS/PCs, we also examined the integration of graft‐derived neurons into host tissues. To evaluate the capacity of graft‐derived neurons to integrate with the host neuronal circuitry, immunostaining was performed using antibodies against HNA, βIII‐tubulin, mouse‐specific Bassoon (Bsn), and human‐specific synaptophysin (hSyn), a presynaptic marker. βIII‐tubulin^+^/HNA^+^ transplanted cell‐derived neurons colocalized with Bsn^+^ synaptic boutons of host neurons (Figure S5A), while βIII‐tubulin^+^/HNA^−^ host mouse neurons colocalized with hSyn^+^ synaptic boutons of transplanted cell‐derived neurons (Figure S5B). These results indicate that the transplanted cell‐derived neurons integrated with host neuronal circuits and formed synapses. Furthermore, since reticulospinal tract (RtST) is known as a spinal cord tract that plays an important role in the initiation of locomotion and postural control,^60^, ^61^ we evaluated RtST fiber regeneration from the brain stem. The BDA‐mediated consecutive neuronal fiber tracing was performed 91 days after transplantation (Figure S5C). The ffiPSC‐gNS/PCs group tended to have larger BDA‐labeled RtST areas compared with the PBS group, but there was almost no significant difference between the two groups (Figure S5D), indicating that the regeneration of neuronal circuits of RtST was less likely to be triggered by transplantation of gNS/PCs.

### 3.5 Transplanted ff‐hiPSC‐gNS/PCs enhance functional recovery following SCI

**FIGURE 6:**
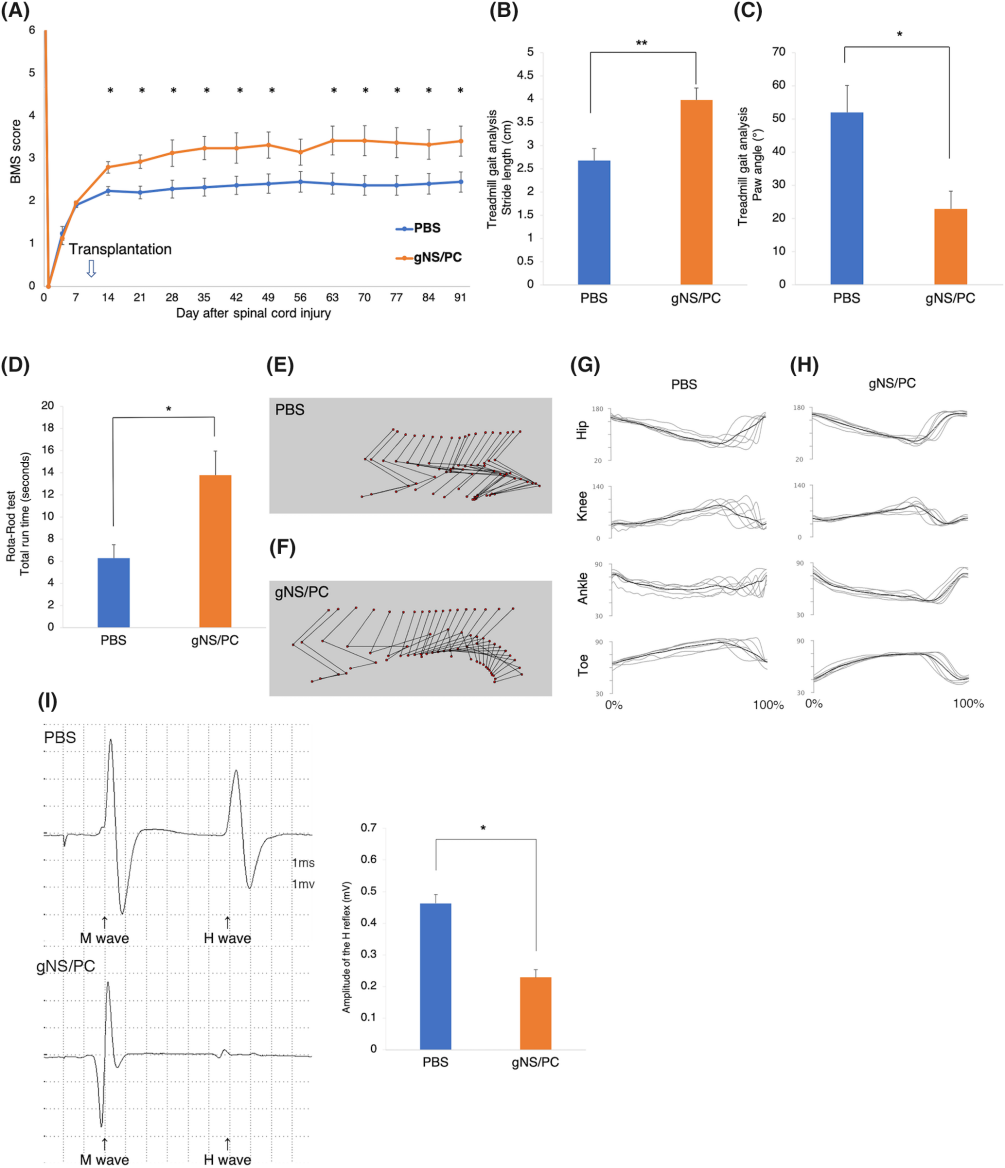
Motor function analyses after transplantation of feeder‐free hiPSCs (ffiPSC)‐gNS/PCs. A, Comparison of Basso Mouse Scale (BMS) scores between the PBS and ffiPSC‐gNS/PCs groups. The BMS scores showed significantly functional recovery in the ffiPSC‐gNS/PCs group compared with that in the PBS group at 14 days after SCI and thereafter. Values are means ± SEM (control group, n = 12; ffiPSC‐gNS/PCs group, n = 14; *P < .05). B,C, Comparison of stride lengths (B) and paw angles (C) between the PBS and ffiPSC‐gNS/PCs groups. Treadmill gait analysis was performed at 12 weeks after transplantation using the DigiGait system. Stride length and stance angle were significantly longer and more parallel, respectively, in the ffiPSC‐gNS/PCs group than in the PBS group. Values are means ± SEM (control group, n = 12; ffiPSC‐gNS/PCs group, n = 14; *P < .05 **P < .01). D, Comparison of rotarod test results between the PBS and ffiPSC‐gNS/PCs groups. The rotarod test was performed at 12 weeks after transplantation. The ffiPSC‐gNS/PCs group ran on the rod for significantly longer than the PBS group. Values are means ± SEM (control group, n = 12; ffiPSC‐gNS/PCs group, n = 14; *P < .05). E,F, Representative kinematics stick diagrams at 12 weeks after transplantation. G,H, Mean (SD) waveforms of hip (Hip), knee (Knee), ankle (Ankle), and toe (Toe) joint angle during treadmill locomotion. I, Comparison of H‐reflex between the PBS and ffiPSC‐gNS/PCs groups using electrophysiological analyses at 12 weeks after transplantation. The amplitude was significantly smaller in the ffiPSC‐gNS/PCs group than in the PBS group. Values are means ± SEM. gNS/PCs, neural stem/progenitor cells with gliogenic competence; PBS, phosphate‐buffered saline

Finally, recovery of locomotor function was evaluated by the BMS score,^31^ rotarod test,^58^, ^62^ treadmill gait analysis using the DigiGait system and kinematic analysis. The BMS score showed significantly improved motor function in the ffiPSC‐gNS/PCs group compared with that in the control PBS group at 14 days after SCI, and functional recovery was significant thereafter (Figure 6A). Body weight was also significantly increased in the ffiPSC‐gNS/PCs group (Figure S3C), consistent with a previous report.^63^ The gait performance of the mice in the two groups was examined using the rotarod test and the DigiGait system at 12 weeks after transplantation. In the ffiPSC‐gNS/PCs group, treadmill gait analysis showed a significantly longer stride length and smaller paw angle (Figure 6B,C). The mice transplanted with the NS/PCs ran on the rotating rod for a significantly longer time than those given PBS (Figure 6D). To evaluate the gait and movement of each joint in more detail, kinematic analysis was performed at 12 weeks after transplantation. Representative stick diagrams of hind limb movements of the swing phase indicate that the ffiPSC‐gNS/PCs group had a smoother step and a more consistent step cycle than the control group (Figure 6E,F). Furthermore, each joint moved with less variation in the ffiPSC‐gNS/PCs group (Figure 6G,H), and various types of joint function in the ffiPSC‐gNS/PCs group were significantly improved compared with those in the control group (Figure S6).

To evaluate the spinal stretch reflex, which plays an important role in locomotion, electrophysiological examination was performed using the H‐reflex at 12 weeks after transplantation. The amplitude of the H‐reflex was significantly lower in the ffiPSC‐gNS/PCs group compared with that in the PBS group (Figure 6I). Together, these results indicate that the gait in the ffiPSC‐gNS/PCs group was also improved compared with that in the control group, suggesting that ffiPSC‐gNS/PCs restore function of residual neuronal fibers to enable precise gait coordination.

## 4 DISCUSSION

Cell replacement has been proposed to treat neurotraumatic injuries, including SCI. In this study, we established robust methods to generate gNS/PCs for treatment of SCI using clinically relevant iPSCs which had been prepared from an HLA‐homozygous super donor. One of the main complications in cell replacement therapy is immune rejection of transplanted allograft cells. Although autologous transplantation is ideal to prevent rejection, clinical grade iPSCs are costly and time consuming to generate and prepare for each patient. Allogenic transplantation using HLA‐matched iPSCs is a more realistic and preferred option for clinical application because immune rejection would be minimal using HLA‐matched donors and patients, as suggested by a non‐human primate study showing that major histocompatibility complex (MHC)‐matching could improve the engraftment of iPSC‐derived neurons.^64^ In Japan, HLA‐A*24:02, HLA‐B*52:01, and HLA‐DRB*15:02 haplotypes are the most frequent (8.5%), and the HLA‐homozygous iPSCs used in the current study matched ~20% of the Japanese population.^65^ Thus, the current procedure to generate gNS/PCs together with iPSCs from a few homozygous donors (super donors) with broad HLA immune‐compatibility would allow for a significant percentage of the population to benefit from iPSC‐based cell therapy.^27^

In addition to the protocols we presented in this study, there are several other procedures available to obtain gliogenic NS/PCs^19^, ^21^, ^22^, ^66^, ^67^ (Table S1). Although we did not directly compare these protocols, our protocol has certain advantages in terms of the required culture period to obtain MBP^+^ oligodendrocytes and higher yield to obtain glial cells.

After transplantation, gNS/PCs differentiated into three neural lineages, including neurons, astrocytes, and oligodendrocytes (Figure 4E). As for oligodendrocyte differentiation, we observed contribution of gNS/PCs to remyelination in injured spinal cord (Figure 5D‐F). It is worth noting that gNS/PCs‐derived OLIG2^+^ GST‐π^−^ immature oligodendrocytes were still present among the grafts (Figure 5A,B). Given that OLIG2 is indicative of migrating oligodendrocytes^68^ and that 77% of cells in the oligodendrocyte lineages still expressed OLIG2 (Figure 5B) at 12 weeks after transplantation, the gNS/PCs‐derived cells that had entered oligodendrocyte lineages still theoretically harbor the potential to promote functional recovery by further remyelination. Oligodendrocyte precursors migrate toward demyelinated lesions^69^; therefore, the residual OLIG2^+^ cells would remyelinate the demyelinated axons over time. Further functional recovery would be enhanced in SCI animal models by rehabilitation, possibly through on‐going myelination together with continued axonal regeneration and synaptogenesis as previously described.^70^ There is a series of reports demonstrating restoration and/or reorganization of neuronal circuits by graft‐derived neurons in SCI animals after NS/PC‐transplantation.^71^ Although we did not observe significant regeneration of the RtST, gNS/PCs still hold the capacity of neuronal differentiation and functional motor recovery might be acquired by neurons differentiated from gNS/PCs (Figure 4D). Indeed, we observed synapse formation between host and gNS/PCs‐derived neurons (Figure S5A,B), providing mechanistic insight into the functional recovery observed after transplantation. Taken together, remyelination of damaged tissues in addition to neuronal replacement would be mediated by gNS/PCs to enhance functional recovery of SCI after transplantation.

One might ask whether gNS/PCs exerted functional recovery more efficiently than neurogenic NS/PCs. In our previous reports using integration‐free iPSCs,^34^ functional recovery after transplantation was not obvious using neurogenic NS/PCs, indicating that gNS/PCs are more beneficial for SCI patients in clinical settings. Indeed, we transplanted nNS/PCs derived from same parental iPSCs and did not observe functional recovery after the transplantation, indicating that gNS/PCs are superior cell sources for cell‐based therapy for SCI (Figure S7). Importantly, this observation might be supported by the previous finding using directly reprogrammed NS/PC from somatic cells.^9^ They also found more beneficial aspects of NS/PCs which had capacity to differentiate into oligodendrocytes. To acquire cell source for transplantation via directly reprogramming approach might be more realistic in the setting of autologous transplantation. Although, it would be challenging to prepare large amounts of cells with quality assurance for autologous without bypassing iPSCs, cell‐therapy relying on NS/PCs with gliogenic differentiation capacity hold promising therapeutic potential. However, gNS/PCs‐derived oligodendrocytes still need residual host neurons, which are demyelinated upon SCI. Therefore, criteria to identify SCI patients who will display functional recovery with gNS/PCs should be determined and examined in future studies.

The current study has several limitations that should be discussed. First, although iPSCs provided by CiRA were generated and maintained under good manufacture practice (GMP)‐conditions,^27^ the current condition to generate gNS/PCs are not xeno‐free, which does not fulfill the criteria for GMP‐quality. To meet the GMP‐grade culture condition, several supplements utilized in our procedures including B27 should be replaced with GMP‐grade xeno‐free products. This issue should be overcome to produce clinically relevant iPSC‐derived cell products. Second, we used iPSC lines that are homozygous for the most frequent HLA haplotype. A previous study estimated that iPSCs with 50 unique homozygous HLA haplotypes would match ~90% of the Japanese population.^72^ Thus, future studies need to validate whether the current approach to generate gNS/PCs is applicable across different HLA‐homozygous iPSCs. Third, although we observed functional recovery, the most important cell types for functional recovery of SCI after transplantation are still unclear. Future studies should seek to develop a procedure to bias NS/PCs toward selected cell lineages that exhibit greater therapeutic potential against neurotraumatic injuries.

## 5 CONCLUSION

We established a robust protocol to generate gNS/PCs from clinically relevant iPSCs. The gNS/PCs derived from HLA‐homozygous iPSCs exhibited prominent favorable outcomes after transplantation into an SCI animal model, providing a promising cell source for treating SCI in future clinical settings.

## CONFLICT OF INTEREST

H.O. is a compensated scientific consultant of San Bio, Co., Ltd., and K Pharma, Inc. and has received research funding from Dainippon Sumitomo Pharmaceutical Co.Ltd. M.N. is a compensated scientific consultant of K Pharma, Inc. M. Isoda and M. Inoue are employed by Sumitomo Dainippon Pharma. The other authors declared no potential conflicts of interest.

## AUTHOR CONTRIBUTIONS

Y.K., M. Isoda: conception and design, collection and assembly of data, data analysis and interpretation, manuscript writing; T. Sanosaka: collection and assembly of data, data analysis and interpretation; R.S., S.I., T.O., M.S., M. Inoue: data analysis and interpretation; I.K., S.S., T. Shindo: collection and assembly of data; M.M., M.N., H.O.: reviewing and editing the manuscript; N.N.: reviewing and editing the manuscript, final approval of manuscript; J.K.: conception and design, financial support, administrative support, collection and assembly of data, data analysis and interpretation, manuscript writing, final approval of manuscript.

## Supporting information


**Appendix** **S1**: Supplementary Experimental procedures

**Figure S1** **Neurogenic NS/PCs generated from ffiPSCs**
Schematic illustration of the protocol for generating neurogenic NS/PCs from hiPSCs (WJ14s01) with representative images of differentiation capacity. S, SB431542; L, LDN193189. Differentiation of the NS/PCs was examined using differentiation markers for neurons (βIII‐tubulin: magenta), astrocytes (GFAP: red) and oligodendrocytes (O4: green). Scale bar, 100 μm.

**Figure S2** **Comparison of Regional identity of NS/PCs derived from ffiPSCs**
**A)** Schematic illustration of regional genes from telencephalon to spinal cord.
B) Boxplot demonstrating regional gene expression in ffiPSC‐nNS/PCs and ffiPSC‐gNS/PCs (n = 91 and 150 sample points, respectively).

**Figure S3** **Transplanted ffiPSC‐gNS/PCs suppress spinal cord atrophy and body weight loss**
A and B) Representative H&E‐stained images of axial sections at the lesion epicenter and at sites 4 mm rostral and caudal in the PBS and the ffiPSC‐gNS/PCs groups. Scale bars, 200 μm. Quantitative analysis of the spinal cord revealed that the area of axial sections at the lesion epicenter was significantly larger in the ffiPSC‐gNS/PCs group than in the PBS group. Values are means ± SEM (control group, n = 6; ffiPSC‐gNS/PCs group, n = 6; P** < 0.01).
C) Comparison of body weight gain and loss (body weight±) from day 7 after SCI to 12 weeks after transplantation among the PBS and ffiPSC‐gNS/PCs groups. The body weight ± was significantly higher in the ffiPSC‐gNS/PCs group than in the PBS group. Values are means ± SEM (control group, n = 12; ffiPSC‐gNS/PCs group, n = 14; *P < 0.05).

**Figure S4** **Transplanted ffiPSC‐gNS/PCs reinforce MBP** ^**+**^ **areas**
A) Representative images of axial sections stained for MBP. Scale bar, 50 mm.
B) Comparison of MBP^+^ areas among the PBS and the ffiPSC‐gNS/PCs groups at 12 weeks after transplantation. The MBP^+^ areas were significantly larger in the ffiPSC‐gNS/PCs group than in the PBS group. Values are means ± SEM (control group, n = 6; ffiPSC‐gNS/PCs group, n = 6; *P < 0.05).

**Figure S5** **Transplanted ffiPSC‐gNS/PCs contribute to neuronal relay by synapse formation with host mouse neurons**
A and B) Representative images of immunohistochemistry using antibodies for HNA, βIII‐tubulin, and the mouse presynaptic marker Bsn or the human‐specific presynaptic marker hSyn. A) indicate that Bsn boutons were apposed to βIII‐tubulin^+^/HNA+ grafted cell‐derived neurons. B) indicate that hSyn boutons were apposed to βIII‐tubulin^+^/HNA^−^ host neurons. Scale bar, 20 mm.
C) Representative images of immunohistochemical staining for BDA‐labeled RtST fibers in axial spinal cord sections at a 4‐mm rostral site.
D) Comparison of BDA^+^ areas among the PBS and the ffiPSC‐gNS/PCs groups at 13 weeks after transplantation. The RtST^+^ areas were significantly larger in the ffiPSC‐NS/PC group than in the PBS group at only 3 mm rostral from the epicenter. There was no significant difference between the two groups at other sites. Values are means ± SEM (control group, n = 6; ffiPSC‐gNS/PCs group, n = 6; *P < 0.05).

**Figure S6** **Kinematic analyses after ffiPSC‐gNS/PCs transplantation**
A‐K) Parameters of kinematic analyses with significant differences. Values are means ± SEM (control group, n = 5; ffiPSC‐gNS/PCs group, n = 8; *P < 0.05).

**Figure S7** **Motor function analyses after transplantation of ffiPSC‐nNS/PCs**
Comparison of BMS scores between the PBS and ffiPSC‐nNS/PC groups. There was no significant difference between the two groups. Values are means ± SEM (control group, n = 10; ffiPSC‐nNS/PCs group, n = 9; *P < 0.05).

**Table S1** Comparison of differentiation capacity of iPSC‐derived oligodendrocyte‐containing neural progenitors

## Data Availability

All RNA‐seq data have been deposited with the Gene Expression Omnibus (GEO) database under accession number GSE138426. The data sets generated and analyzed during the current study are available from the corresponding author upon reasonable request.
