# Supplementary material for: A robust culture system to generate neural progenitors with gliogenic competence from clinically relevant induced pluripotent stem cells for treatment of spinal cord injury
Source: Stem Cells Transl Med. 2020 Nov 23;10(3):398–413. doi: 10.1002/sctm.20-0269 (PMC7900588; doi:10.1002/sctm.20-0269)
Supplement: Supplementary file 1 — Appendix S1: Supplementary Experimental procedures [file SCT3-10-398-s001.docx]

**Supplementary Experimental procedures**

**Single cell RNA-Seq**

NS/PCs were dissociated and sorted into a 96-well plate with a flow cytometer SH800 (SONY, Tokyo, Japan) and lysed in single-cell lysis buffer. Transcript amplification was performed as previously described [4]. Amplified cDNA from single cells was processed for library preparation using a Nextera XT Library Preparation Kit (Illumina, San Diego, CA, USA). The sequencing library was analyzed by massively parallel sequencing using a Hiseq 2500 (Illumina). Raw reads were trimmed based on read quality and read length using Trimmomatic (v0.33) software. Trimmed reads were aligned to reference genome hg19 using Sailfish (v0.7.6) with default settings. Samples were filtered with the following parameters and used for analysis: read number > 1 million, aligned rate > 70%, mitochondria derived reads < 30%, and detected gene number > 13,000. Bioinformatics was performed using the Partek Flow single cell module (Partek).

**mRNA-Seq**

Samples for mRNA-seq were prepared using the TruSeq RNA Sample Prep Kit (Illumina) according to the protocol provided by the manufacture. The sequencing library was sequenced on a HiSeq 2500 (Illumina). Base-calling and chastity ﬁltering were performed using Real-Time Analysis Software version 1.18.61 and raw reads were mapped to reference genome hg19 using Sailfish (v0.7.6) with default settings.

Overall gene expression profiles were evaluated using Exatlas (<https://lgsun.irp.nia.nih.gov/exatlas/)>. The criteria for correlation analysis significance was adjusted with a false discovery rate (FDR) < 0.05 and 2-fold change. The extracted data were visualized with Morpheus (<https://software.broadinstitute.org/morpheus)>. The published gene expression profiles of iPSCs, iPSC-neurospheres and fetal neurospheres were loaded from GSE76900. To evaluate global gene expression transition in ffiPSC-gNS/PCs (passage 2 and 3), genes differentially expressed by 1.5-fold were extracted and GO analyzed using DAVID Bioinformatics Resources (http://david. ncifcrf.gov).

**Immunocytochemistry**

gNS/PC spheres and gNS/PC sphere-derived differentiated cells were fixed in 4% paraformaldehyde (PFA) for 25 min at room temperature and washed three times in PBS. The gNS/PC spheres were subsequently embedded in Optimal Cutting Temperature compound (Sakura Finetechnical) and sectioned at 10 μm on a cryostat (Leica CM3050 S, Leica Microsystems, Wetzlar, Germany). The cells were blocked with PBS containing 10% goat serum for 1 h at room temperature and incubated at 4°C overnight with primary antibodies diluted in PBS containing 10% goat serum. After washing with PBS, cells were incubated for 1 h at room temperature with secondary antibodies diluted in PBS containing 10% goat serum. The following primary antibodies were used for immunofluorescence: anti-OLIG2 (1:300; Millipore, USA; MABN50), anti-PDGFRα (1:300; Cell Signaling Technology; 3174), anti-nELAVL (1:100; Thermo Fisher Scientific, Waltham, MA, USA; A-21271), anti-GFAP (1:1000; Thermo Fisher Scientific; 13-0300), anti-NESTIN (1:300; Millipore; MAB5326), anti-βIII-tubulin (1:500; Sigma-Aldrich, St. Louis, MO, USA; T8660), anti-MAP2 (1:500; Sigma-Aldrich; M4403), anti-NFIA (1:100, Sigma-Aldrich; HPA006111), anti-O4 (1:500, Thermo Fisher Scientific; MAB1326), anti-galactocerebroside (GalC; 1:1000; Millipore; MAB342), and anti-MBP (1:1000; AbD Serotec; MCA409S). Alexa Fluor secondary antibodies (Thermo Fisher Scientific) were used at 1:1000. Cell nuclei were counterstained with 1 μg/ml Hoechst 33342 (DOJINDO). Images were acquired using a fluorescence microscope (BZ-X710; Keyence Co., Osaka, Japan) or a confocal laser scanning microscope (LSM880; Carl Zeiss). For the quantification of OLIG2^+^ and NFIA^+^ cells in gNS/PC spheres, stained images of six spheres were analyzed with BZ-X Viewer software (Keyence). For the quantitative analysis of O4^+^, GFAP^+^, and βIII-tubulin^+^ cells, over 20000 immunostained cells per sample were imaged by acquiring 6 fields of view using the 10x objective and analyzed using the Developer Toolbox software (version 1.9.2; GE Healthcare). First, Hoechst-stained nuclear segmentations were performed and cell intensity segmentation was performed on the channel corresponding to the cytoplasm staining. The sieve operation was applied to remove debris and obtained optimal outlines of desired objects. The number of positive cells were determined according to the overlap of nuclei staining and cytoplasm staining. The percentage of each positive cells to total nuclei counts was calculated.

**Animals**

All experiments were conducted in accordance with the Guidelines for the Care and Use of Laboratory Animals of Keio University and the NIH Guide for the Care and Use of Laboratory Animals. The experimental protocols of the SCI model were approved by the Animal Care Committee at the Keio University School of Medicine (Tokyo, Japan, Permit Number: 13020). The mice were housed 3–5 per cage in a sterile facility under a 12-h light/dark cycle with free access to food and water. The experimental protocols of intracerebral transplantation were approved by the Institutional Animal Care and Use Committee of Sumitomo Dainippon Pharma Co., Ltd. (Kobe, Japan, Permit Number: AN12332).

**Behavioral analysis**

Open field hindlimb locomotor function was evaluated for 12 weeks after transplantation using the Basso Mouse Scale. Well-trained observers who were blind to groupings performed the behavioral analyses. At 12 weeks after transplantation, motor coordination was measured using a rotating rod apparatus (rotarod, Muromachikikai), which consisted of a plastic rod (3 cm diameter and 8 cm long) with a gridded surface. Each mouse was placed on the rod while it rotated at 20 rotations per minute (rpm). A treadmill gait analysis was performed using the DigiGait system (Mouse Specifics, Quincy, MA, USA). The stride length and stance angle were determined on a treadmill set to a speed of 7 cm/s. Kinematics was performed by recording the gait using a camera (Go Pro HERO5 Black CHDX-502). The shoulder, hip, knee, and toe were labeled. Kinematics recording was analyzed using KinemaTracer software (KISSEI COMTEC).

**Electrophysiology**

Electrophysiological experiments were performed using a Neuropack S1 MEB-9402 (Nihon Kohden, Tokyo, Japan) as previously described [5] at 12 weeks after transplantation. Mice were anesthetized using only ketamine (120 mg/kg). Mouse forelimbs and hindlimbs were fixed with tape on a Styrofoam plate. Tibial nerve stimulation was performed using two needle electrodes. The stimulating electrodes were placed inside and outside plantar muscle, and the ground electrode was placed in the animal's ankle. Electrode positioning was performed under a microscope. Single biphasic pulses with an intensity of 2-5 mA, a duration of 0.2 ms, and an interstimulus interval of 1 Hz was used.

**Histological analysis**

All mice were anesthetized and euthanized by transcardial perfusion of 0.1 M PBS containing 4% PFA at 12 weeks after transplantation. Dissected spinal cords were embedded in Optimal Cutting Temperature compound (Sakura Finetechnical Co.) and sectioned in the sagittal/axial plane at a thickness of 14/16 μm on a cryostat (Leica CM3050 S, Leica Microsystems). Spinal cord sections were histologically evaluated by hematoxylin & eosin and Luxol fast blue staining and by immunohistochemistry. Tissue sections were stained with the following primary antibodies: anti-pan-ELAVL(Hu) (human IgG, 1:1000, a gift from Dr. Robert Darnell; The Rockefeller University, New York, NY, USA), anti-GFAP (mouse IgG2b, 1:5000; Abcam, Cambridge, UK, ab10062), anti-APC (mouse IgG2b, 1:300; Abcam, ab16794), anti-human-specific NESTIN protein (rabbit IgG, 1:200; described previously [6]), anti-Ki67 (Ki67; rabbit IgG, 1:1000; Leica Biosystems, Wetzlar, Germany), anti-HNA (mouse IgG, 1:100; Chemicon, Temecula, CA, USA, MAB4383), anti-human cytoplasm (STEM121; mouse IgG1, 1:300; Takara Bio, Kusatsu, Japan, Y40410), anti-OLIG2 (goat IgG, 1:100; R&D systems, AF2418), anti-GST-π (rabbit IgG, 1:500; MBL, 312), anti-βIII-tubulin (mouse IgG, 1:300; Sigma-Aldrich, T8660), anti-human tau (mouse IgG, 1:500; Thermo Fisher Scientific, MN1000), anti-MBP (rat IgG, 1:500; Bio-rad, MCA409S), anti-BSN (mouse IgG2a, 1:200; GeneTex, GTX13249), and anti-hSyn (Mouse IgM, 1:200; Millipore, MAB329).

To quantify the proportion of each cellular phenotype in vivo, samples were visualized using a fluorescence microscope (BZ-X710; Keyence) or a confocal laser scanning microscope (LSM 700, Carl Zeiss, Jena, Germany). We randomly selected and captured ten regions within 4 mm rostral and caudal to the lesion epicenter at 63× primary objective. The numbers of marker-positive cells, such as HNA^+^, Ki67^+^, OCT3/4^+^, NESTIN^+^, Hu^+^, GFAP^+^, APC^+^, OLIG2^+^, and GST-π^+^ cells, as well as BDA-labeled RtST^+^ fibers were counted in each section (n = 6 per group).

For lesion morphometry, axial serial H&E stained sections were used. We performed H&E analyses on tissue ±4 mm the epicenter (n = 6/group). Images were captured for tissue sections every 250 μm on an fluorescence microscope (BZ-X710; Keyence) at 10× primary objective.

To quantify myelinated area of the spinal cord around lesion area, axial serial LFB and MBP stained section were used. We performed them on tissue ±1 mm the epicenter (n = 6/group). Images were captured for tissue sections every 250 μm on an fluorescence microscope (BZ-X710; Keyence) at 10× primary objective.

Grafted rats were deeply anesthetized and transcardially perfused with PBS, followed by 4% PFA. The dissected brains were embedded in Optimal Cutting Temperature compound (Sakura Finetechnical) and sectioned in the coronal plane at a thickness of 5 μm on a cryostat. Immunohistochemistry was performed using the following primary antibodies: anti-Ki67 (rabbit IgG, 1:300; Abcam, ab16667), anti-HNA (mouse IgG, 1:100; Chemicon, MAB4383), and anti-OCT3/4 (1:100, Santa Cruz, sc-5279).

**Immunoelectron microscopy**

The detailed immunoelectron microscopy procedure was described previously [7]. Briefly, spinal cord tissues were perfused and postfixed with 4% PFA for 12 h, followed by cryoprotective treatment with 15% and 30% sucrose. Frozen tissue blocks in cryocompound were sectioned at 20 μm thickness on a cryostat (Leica CM3050S). Sections were incubated with 5.0% Block Ace solution (DS Pharma Biomedical, Japan) containing 0.01% saponin in 0.1 M PB for an hour and then incubated with primary mouse anti-human cytoplasm antibody (1:200, STEM121, Takara Bio) for 72 h at 4°C, followed by incubation with FluoroNanogold-conjugated goat anti-mouse secondary antibody (1:100, Thermo Fisher Scientific, USA) for 24 h at 4°C. After 2.5% glutaraldehyde fixation in 0.1 M PB, nanogold signals were enhanced with R-Gent SE-EM Silver Enhancement Reagents (Aurion) for 30 min at 25°C. Gold-labeled sections were postfixed with 1.0% OsO_4_ for 90 min at 4°C, en bloc stained with uranyl acetate for 20 min at 25°C, dehydrated through a graded ethanol series and embedded into pure Epon. Ultrathin sections (80 nm) were prepared with an ultramicrotome (UC7, Leica) and stained with uranyl acetate and lead citrate. The sections were imaged by transmission electron microscopy (JEM1400 plus, JEOL, Japan) and multibeam scanning electron microscopy (multiSEM505, Carl Zeiss, Germany).

**Anterograde labeling of the RtST**

Mice were anesthetized and placed in stereotaxic head holders. A pressure injection (50 nL) of BDA solution (10,000 MW; Molecular Probes; 10% in distilled water) was performed at the reticular formation (depth 3.4–4.85 mm; lateral 0–1.25 mm; and rostral 5.63–7.19 mm) as described previously [8,9]. Two weeks later, the mice were sacrificed and histological analyses performed.

**Supplementary References**

1 Najm FJ, Zaremba A, Caprariello AV et al. Rapid and robust generation of functional oligodendrocyte progenitor cells from epiblast stem cells. Nature methods 2011;8(11):957-962.

2 Douvaras P, Fossati V. Generation and isolation of oligodendrocyte progenitor cells from human pluripotent stem cells. Nat Protoc 2015;10(8):1143-1154.

3 Gorris R, Fischer J, Erwes KL et al. Pluripotent stem cell-derived radial glia-like cells as stable intermediate for efficient generation of human oligodendrocytes. Glia 2015;63(12):2152-2167.

4 Hayashi T, Ozaki H, Sasagawa Y et al. Single-cell full-length total RNA sequencing uncovers dynamics of recursive splicing and enhancer RNAs. Nat Commun 2018;9(1):619.

5 Piaton G, Aigrot MS, Williams A et al. Class 3 semaphorins influence oligodendrocyte precursor recruitment and remyelination in adult central nervous system. Brain 2011;134(Pt 4):1156-1167.

6 Kanemura Y, Mori H, Kobayashi S et al. Evaluation of in vitro proliferative activity of human fetal neural stem/progenitor cells using indirect measurements of viable cells based on cellular metabolic activity. Journal of neuroscience research 2002;69(6):869-879.

7 Shibata S, Iseda T, Mitsuhashi T et al. Large-Area Fluorescence and Electron Microscopic Correlative Imaging With Multibeam Scanning Electron Microscopy. Front Neural Circuits 2019;13:29.

8 Okubo T, Nagoshi N, Kohyama J et al. Treatment with a Gamma-Secretase Inhibitor Promotes Functional Recovery in Human iPSC- Derived Transplants for Chronic Spinal Cord Injury. Stem cell reports 2018;11(6):1416-1432.

9 Liang H, Watson C, Paxinos G. Terminations of reticulospinal fibers originating from the gigantocellular reticular formation in the mouse spinal cord. Brain structure & function 2016;221(3):1623-1633.
