# Supplementary figures and images for: A robust culture system to generate neural progenitors with gliogenic competence from clinically relevant induced pluripotent stem cells for treatment of spinal cord injury
Source: Stem Cells Transl Med. 2020 Nov 23;10(3):398–413. doi: 10.1002/sctm.20-0269 (PMC7900588; doi:10.1002/sctm.20-0269)

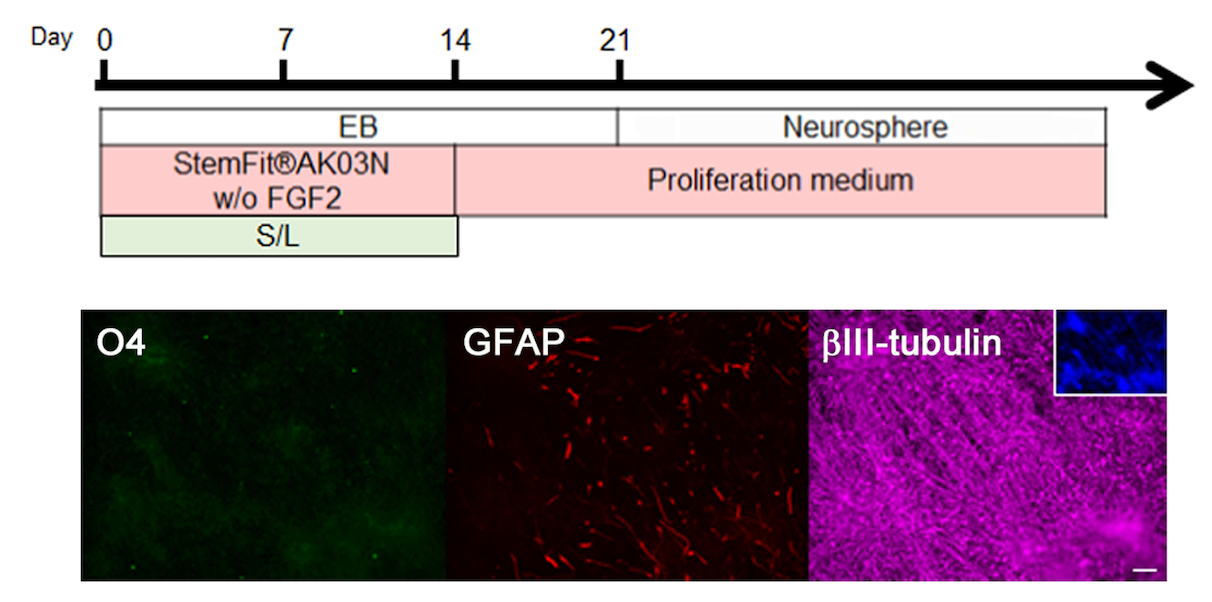

Supplement: Supplementary file 2 — Figure S1 Neurogenic NS/PCs generated from ffiPSCs Schematic illustration of the protocol for generating neurogenic NS/PCs from hiPSCs (WJ14s01) with representative images of differentiation capacity. S, SB431542; L, LDN193189. Differentiation of the NS/PCs was examined using differentiation markers for neurons (βIII‐tubulin: magenta), astrocytes (GFAP: red) and oligodendrocytes (O4: green). Scale bar, 100 μm. [file SCT3-10-398-s002.tif]

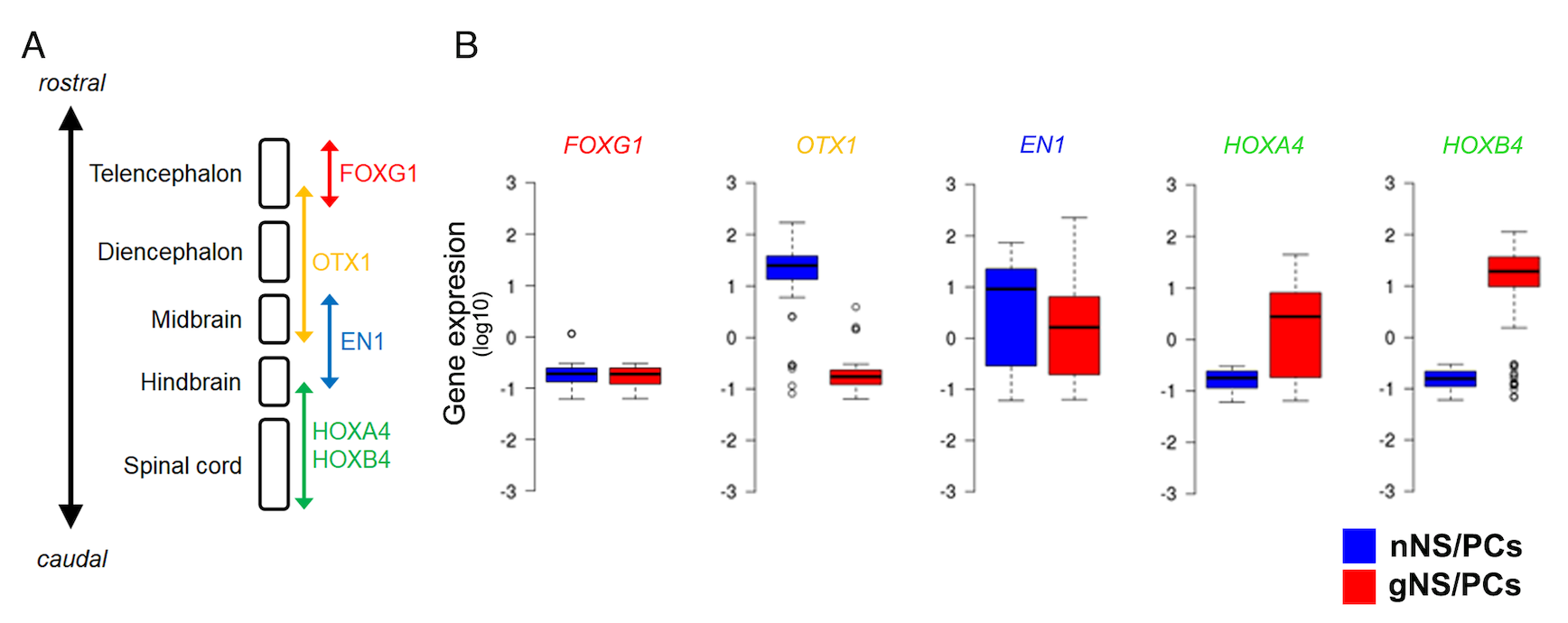

Supplement: Supplementary file 3 — Figure S2 Comparison of Regional identity of NS/PCs derived from ffiPSCs A) Schematic illustration of regional genes from telencephalon to spinal cord. B) Boxplot demonstrating regional gene expression in ffiPSC‐nNS/PCs and ffiPSC‐gNS/PCs (n = 91 and 150 sample points, respectively). [file SCT3-10-398-s003.tif]

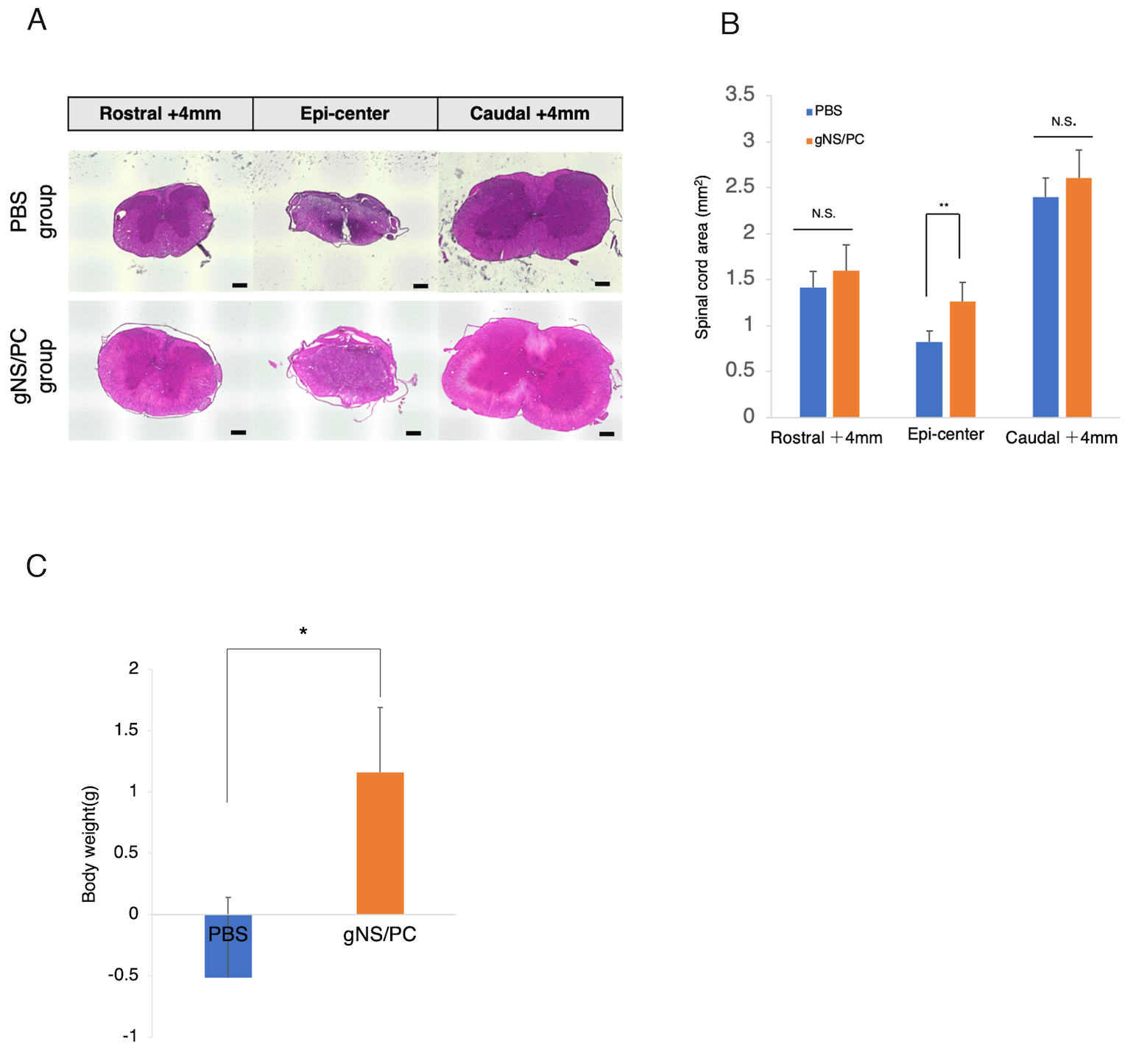

Supplement: Supplementary file 4 — Figure S3 Transplanted ffiPSC‐gNS/PCs suppress spinal cord atrophy and body weight loss A and B) Representative H&E‐stained images of axial sections at the lesion epicenter and at sites 4 mm rostral and caudal in the PBS and the ffiPSC‐gNS/PCs groups. Scale bars, 200 μm. Quantitative analysis of the spinal cord revealed that the area of axial sections at the lesion epicenter was significantly larger in the ffiPSC‐gNS/PCs group than in the PBS group. Values are means ± SEM (control group, n = 6; ffiPSC‐gNS/PCs group, n = 6; P** < 0.01). C) Comparison of body weight gain and loss (body weight±) from day 7 after SCI to 12 weeks after transplantation among the PBS and ffiPSC‐gNS/PCs groups. The body weight ± was significantly higher in the ffiPSC‐gNS/PCs group than in the PBS group. Values are means ± SEM (control group, n = 12; ffiPSC‐gNS/PCs group, n = 14; *P < 0.05). [file SCT3-10-398-s004.tif]

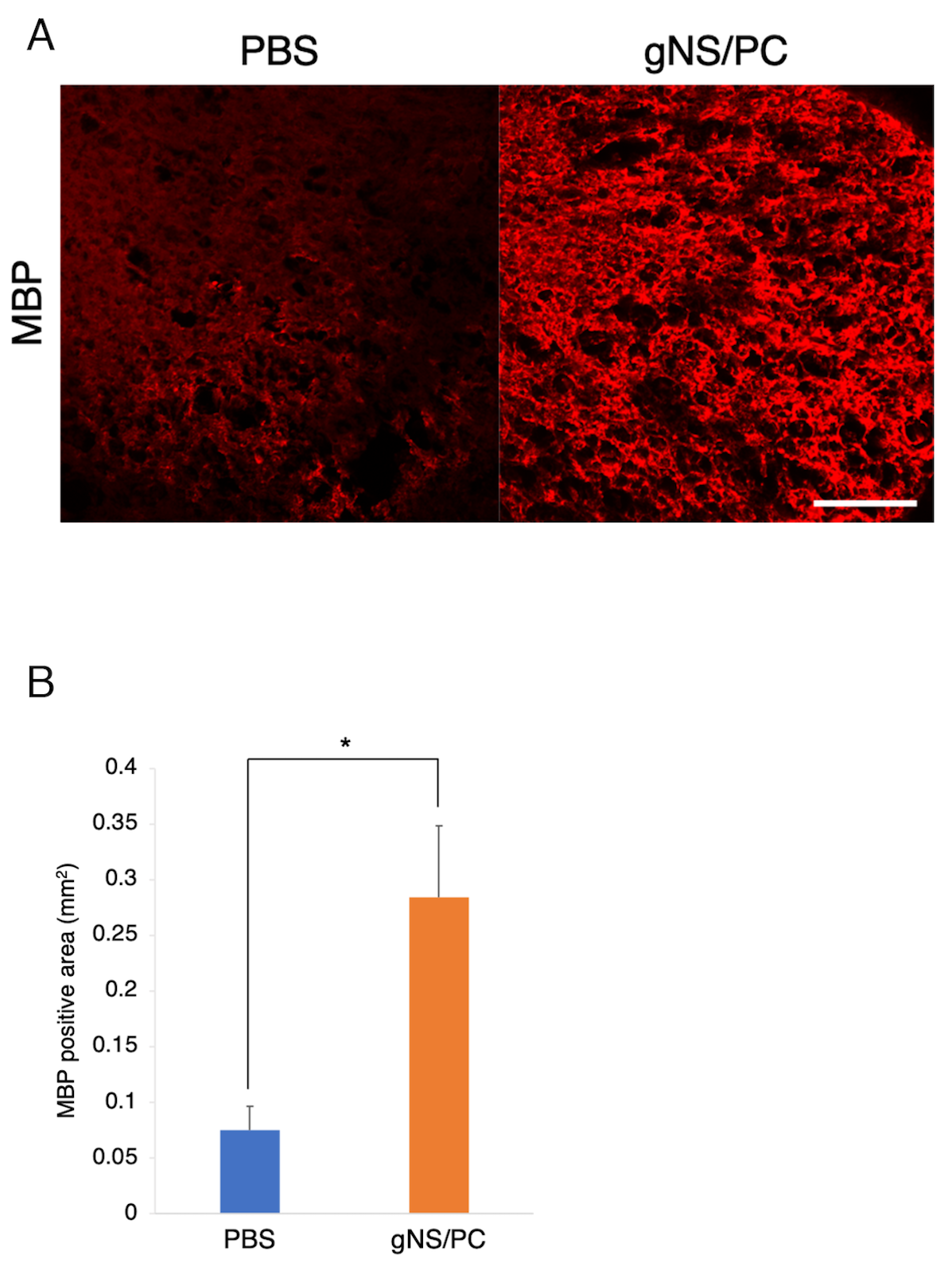

Supplement: Supplementary file 5 — Figure S4 Transplanted ffiPSC‐gNS/PCs reinforce MBP + areas A) Representative images of axial sections stained for MBP. Scale bar, 50 mm. B) Comparison of MBP+ areas among the PBS and the ffiPSC‐gNS/PCs groups at 12 weeks after transplantation. The MBP+ areas were significantly larger in the ffiPSC‐gNS/PCs group than in the PBS group. Values are means ± SEM (control group, n = 6; ffiPSC‐gNS/PCs group, n = 6; *P < 0.05). [file SCT3-10-398-s005.tif]

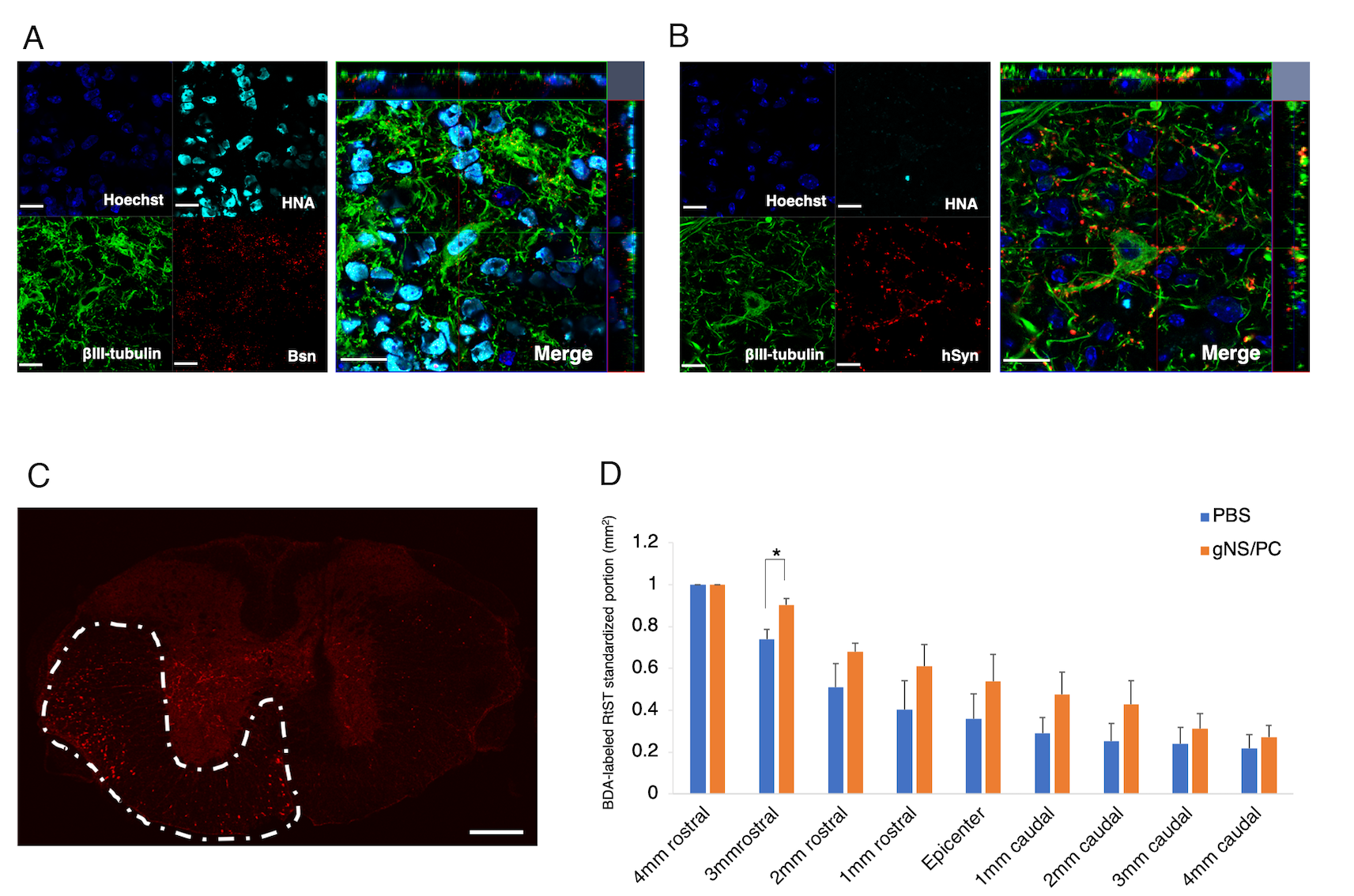

Supplement: Supplementary file 6 — Figure S5 Transplanted ffiPSC‐gNS/PCs contribute to neuronal relay by synapse formation with host mouse neurons A and B) Representative images of immunohistochemistry using antibodies for HNA, βIII‐tubulin, and the mouse presynaptic marker Bsn or the human‐specific presynaptic marker hSyn. A) indicate that Bsn boutons were apposed to βIII‐tubulin+/HNA+ grafted cell‐derived neurons. B) indicate that hSyn boutons were apposed to βIII‐tubulin+/HNA− host neurons. Scale bar, 20 mm. C) Representative images of immunohistochemical staining for BDA‐labeled RtST fibers in axial spinal cord sections at a 4‐mm rostral site. D) Comparison of BDA+ areas among the PBS and the ffiPSC‐gNS/PCs groups at 13 weeks after transplantation. The RtST+ areas were significantly larger in the ffiPSC‐NS/PC group than in the PBS group at only 3 mm rostral from the epicenter. There was no significant difference between the two groups at other sites. Values are means ± SEM (control group, n = 6; ffiPSC‐gNS/PCs group, n = 6; *P < 0.05). [file SCT3-10-398-s006.tif]

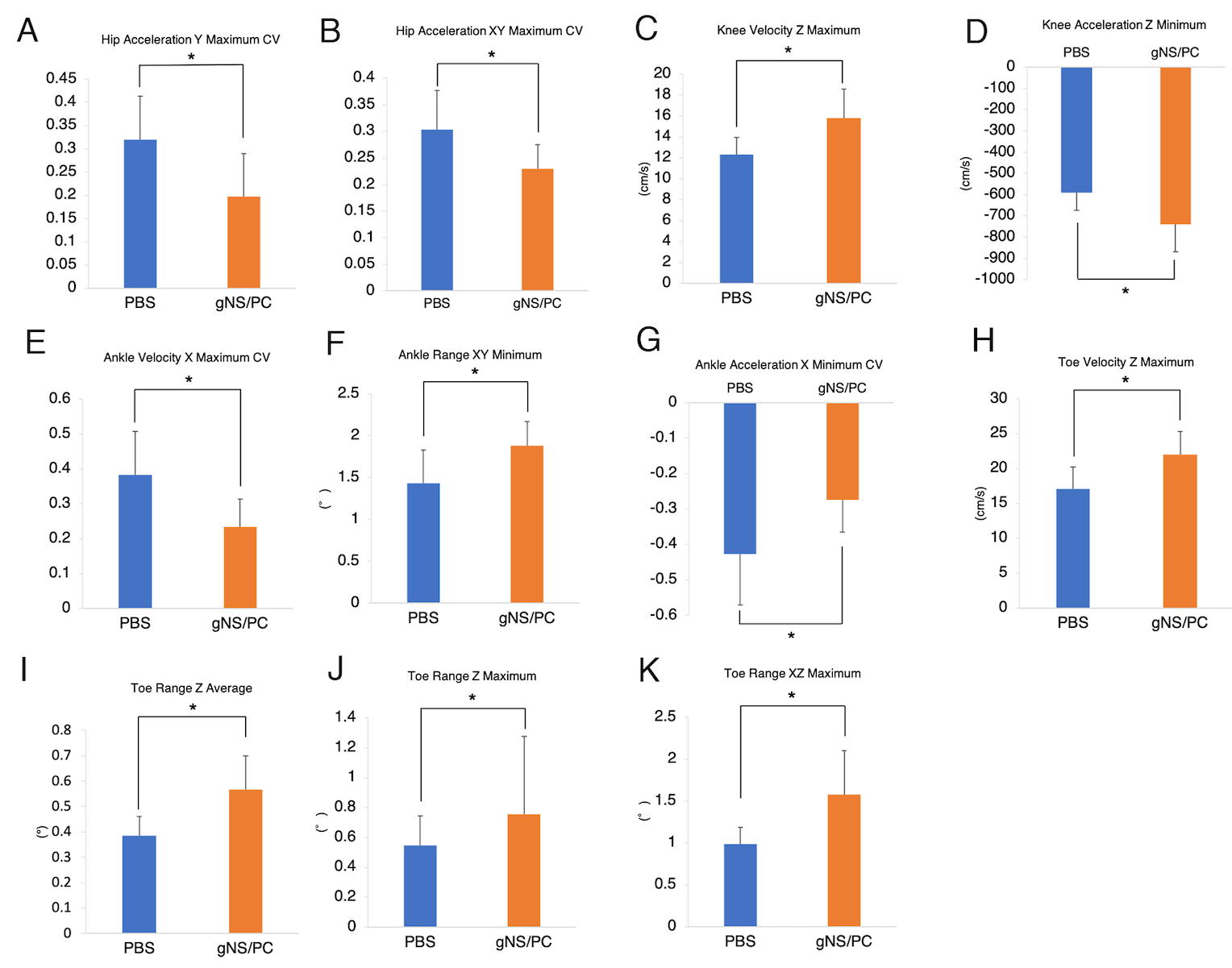

Supplement: Supplementary file 7 — Figure S6 Kinematic analyses after ffiPSC‐gNS/PCs transplantation A‐K) Parameters of kinematic analyses with significant differences. Values are means ± SEM (control group, n = 5; ffiPSC‐gNS/PCs group, n = 8; *P < 0.05). [file SCT3-10-398-s007.tif]

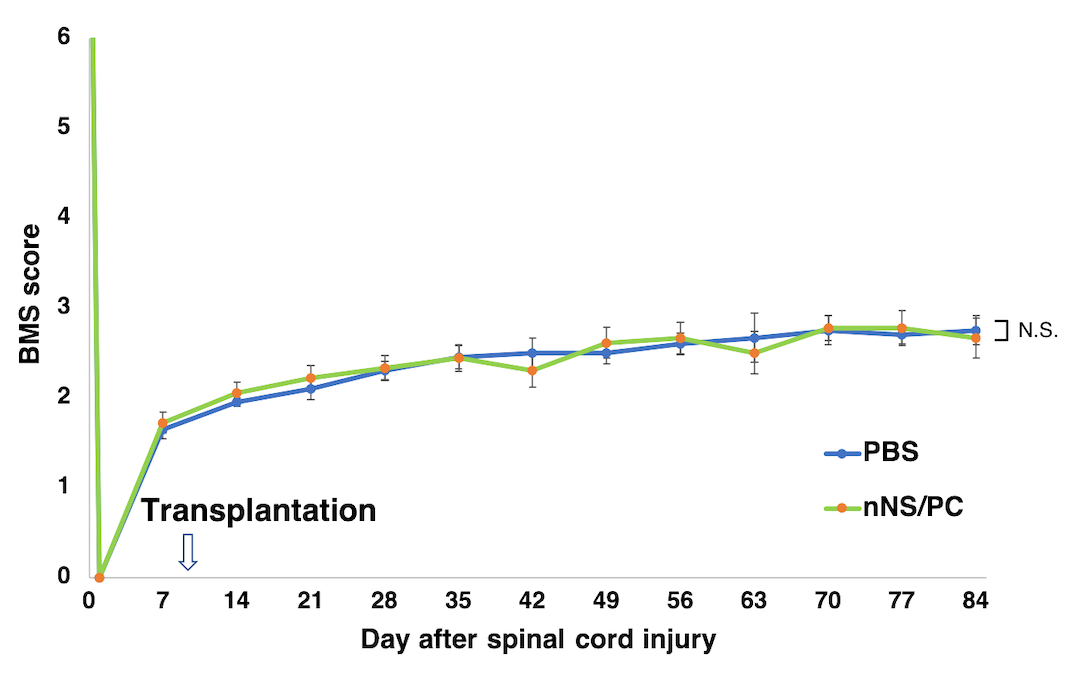

Supplement: Supplementary file 8 — Figure S7 Motor function analyses after transplantation of ffiPSC‐nNS/PCs Comparison of BMS scores between the PBS and ffiPSC‐nNS/PC groups. There was no significant difference between the two groups. Values are means ± SEM (control group, n = 10; ffiPSC‐nNS/PCs group, n = 9; *P < 0.05). [file SCT3-10-398-s008.tif]

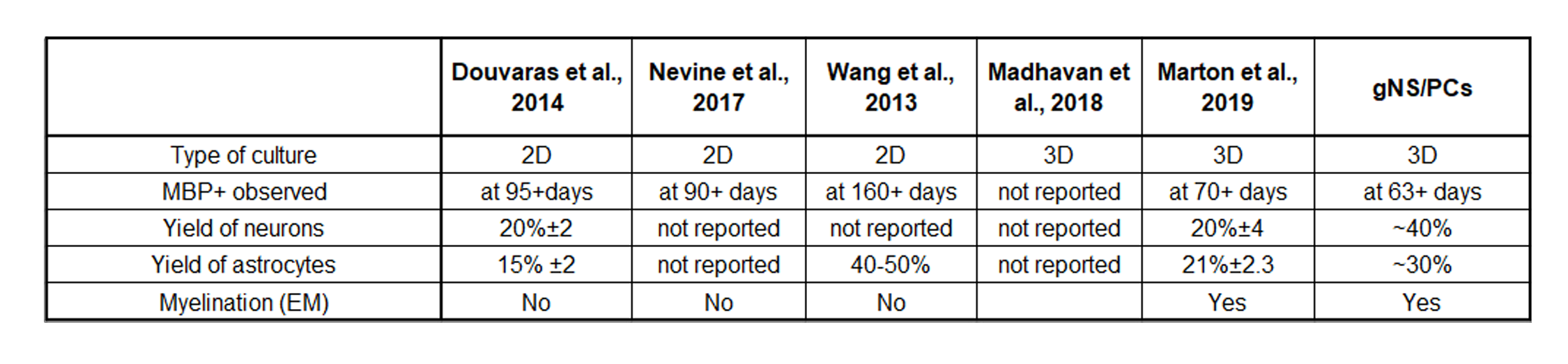

Supplement: Supplementary file 9 — Table S1 Comparison of differentiation capacity of iPSC‐derived oligodendrocyte‐containing neural progenitors [file SCT3-10-398-s009.tif]
